# Long COVID risk factors and outcomes among solid organ transplant recipients: a retrospective cohort study

**DOI:** 10.3389/fsurg.2025.1602167

**Published:** 2025-08-25

**Authors:** Micaela N. Sandoval, Linda W. Moore, Howard J. Huang, Edward A. Graviss

**Affiliations:** ^1^ Department of Epidemiology, The University of Texas Health Science Center School of Public Health, Houston, TX, United States; ^2^ Department of Surgery, Houston Methodist Hospital, Houston, TX, United States; ^3^ Department of Medicine, Houston Methodist Hospital, Houston, TX, United States; ^4^ Department of Pathology and Genomic Medicine, Houston Methodist Research Institute, Houston, TX, United States

**Keywords:** COVID-19, long COVID, solid organ transplant (SOT), epidemiology, infectious diseases

## Abstract

**Background:**

Solid organ transplant (SOT) recipients are not only at increased risk of morbidity and mortality due to acute COVID-19 but may also experience poor long-term outcomes due to post-acute COVID-19 syndromes, including long COVID.

**Methods:**

This retrospective, registry-based chart review evaluated graft failure and mortality among SOT recipients diagnosed with COVID-19 at a large, urban transplant center in Houston, Texas, USA. Patient populations were analyzed separately according to their long COVID status at the time of transplant to preserve the temporal relationship between the exposure (long COVID) and the outcome (graft failure or mortality).

**Results:**

In total, 146 (5%, 146/3,202) patients were diagnosed with long COVID, 443 (14%, 443/3,202) patients expired during the study period, and 202 (6%, 202/3,202) were diagnosed with graft failure. Overall, patients with long COVID were older, had an increased comorbidity burden, and were more likely to be lung, heart, or heart–lung recipients compared with those who were not diagnosed with long COVID. Long COVID was not significantly associated with death or graft failure in this study population, though relationships varied across subpopulations.

**Conclusions:**

The observed differences between patients diagnosed with COVID-19 and long COVID before and after transplant warrant additional studies as the proportion of people with some SARS-CoV-2 infection history approaches 90%. Future investigations may prioritize longitudinal follow-up of long COVID patients diagnosed before or after transplant to determine specific etiologies of long-term morbidity and mortality.

## 1 Background

Post-COVID conditions, including “long COVID,” have emerged as significant health threats, with patients experiencing debilitating symptoms and decreased quality of life, months to years following even mild COVID-19 episodes (1–5). The etiology of long COVID is still being uncovered, but possible subtypes include persistent viral infection, chronic inflammatory response, and lasting organ damage from the original SARS-CoV-2 infection (6–9). Even in the post-COVID vaccination era, long COVID is becoming an important public health issue, with studies estimating up to 1 in 10 adults in the USA have experienced at least one long COVID symptom (10).

The immune dysfunction and organ damage associated with long COVID are especially concerning in high-risk populations, such as solid organ transplant (SOT) recipients. SOT recipients, who have undergone immunosuppressive treatment and carry increased comorbidity burdens, have an increased risk of morbidity and mortality due to acute COVID-19 (11–15). Additionally, SOT recipients may produce poor humoral and cellular immune responses to SARS-CoV-2 vaccination, which could result in more severe COVID-19 infections (16–19). Investigations into the long-term outcomes associated with post-COVID conditions among SOT recipients are ongoing, but early results indicate post-acute COVID symptoms are highly prevalent in this population (20–23).

The association between acute COVID-19, long COVID, and SOT is multifaceted, as patients with severe COVID-19 may require organ transplants (24, 25), and SOT recipients may be more susceptible to morbidity and mortality in both the acute and post-acute phases of COVID-19. This complex temporal relationship is further muddled by the overlap between long COVID symptoms and conditions associated with SOT. This investigation evaluated graft failure and mortality among SOT recipients diagnosed with COVID-19 at a large, urban transplant center in Houston, Texas, USA.

## 2 Materials and methods

A retrospective, registry-based chart review was conducted investigating long COVID as a risk factor for all-cause mortality and graft failure among solid organ transplant (SOT) recipients diagnosed with COVID-19 in a large metropolitan healthcare system in Houston, Texas, USA. Electronic health record data and United Network for Organ Sharing (UNOS) transplant tracking data were collected from consecutive SOT recipients who received a diagnostic SARS-CoV-2 test between 1 March 2020 and 15 December 2024. The UNOS system, which collects active follow-up data from all patients transplanted within this healthcare system, is the most complete death and graft failure dataset available; patients lost to UNOS are considered lost to follow-up and censored or excluded as appropriate. An additional reference population of patients who received SOT within the same healthcare system but did not have a reported COVID-19 episode during the study period was analyzed using the site-specific UNOS dataset. Patients who received only bone marrow, corneal, or stem cell transplants and those who received only autograft transplants were excluded from all analyses. The date of transplant for patients transplanted before the study period was recorded as the date of the most recently transplanted organ still active at the beginning of the study period, 1 March 2020. For patients who received their first eligible transplant during the study period, the first transplant date was used. Each patient contributed only one transplant episode; re-transplants were not considered in this analysis. Patients who were no longer being followed by UNOS at the study start and who did not receive any subsequent transplants within the study period were excluded from all analyses.

### 2.1 Study exposures

The primary exposure was long COVID status determined by the presence of the ICD-10 code U09.9, which was initiated on 1 October 2021 (26). Centers for Disease Control and Prevention (CDC) defines long COVID as “a chronic condition that occurs after SARS-CoV-2 infection and is present for at least 3 months” (27). Demographic and clinical data were collected via the COVID-19 Surveillance and Outcomes Registry (CURATOR), a COVID-19-specific electronic health records (EHR) research, surveillance, and analysis project (28). All-cause hospitalization and intensive care admissions within 30 days of COVID-19 diagnosis were recorded. SOT details, including date of transplant, organ, donor type (deceased vs. living), and dates of follow-up, were retrieved from the UNOS dataset. Clinical history was characterized using diagnosis codes (ICD-10); the Charlson comorbidity index was additionally calculated as a measure of overall comorbidity burden (29–31). Patients were classified as COVID-19 cases if they received a positive diagnostic result from a SARS-CoV-2 RNA polymerase chain reaction (PCR) assay or a viral antigen assay at Houston Methodist during the study period. COVID-19 case status was recorded on the date of the first reported positive SARS-CoV-2 result. The date of the first recorded COVID-19 episode was categorized according to waves in COVID-19 incidence across Texas to reflect local epidemic dynamics and approximate exposure across variants (32). The area deprivation index (ADI), which measures relative deprivation between all census block groups by state (33, 34), was calculated from the geocoded patient-provided home addresses at the time of the first COVID-19 episode. We were not able to include COVID-19 vaccination status in this analysis due to poor data capture. Transplant patients in the study setting became eligible for COVID-19 mRNA vaccines in December 2020, and transplant-specific vaccination programs utilizing the NT162b2 (Pfizer-BioNTech) or the mRNA-1273 (Moderna) began in January 2021. The specific vaccine each patient received was based on health care and public health procurement.

### 2.2 Outcomes

A composite outcome of all-cause mortality or graft failure was the primary endpoint; the date of the first recorded event was used as the outcome date. Patients were considered lost to follow-up if they were no longer being followed by UNOS for any reason other than death or graft failure. The primary cause of death was extracted from the UNOS dataset.

### 2.3 Analysis

**Figure 1:**
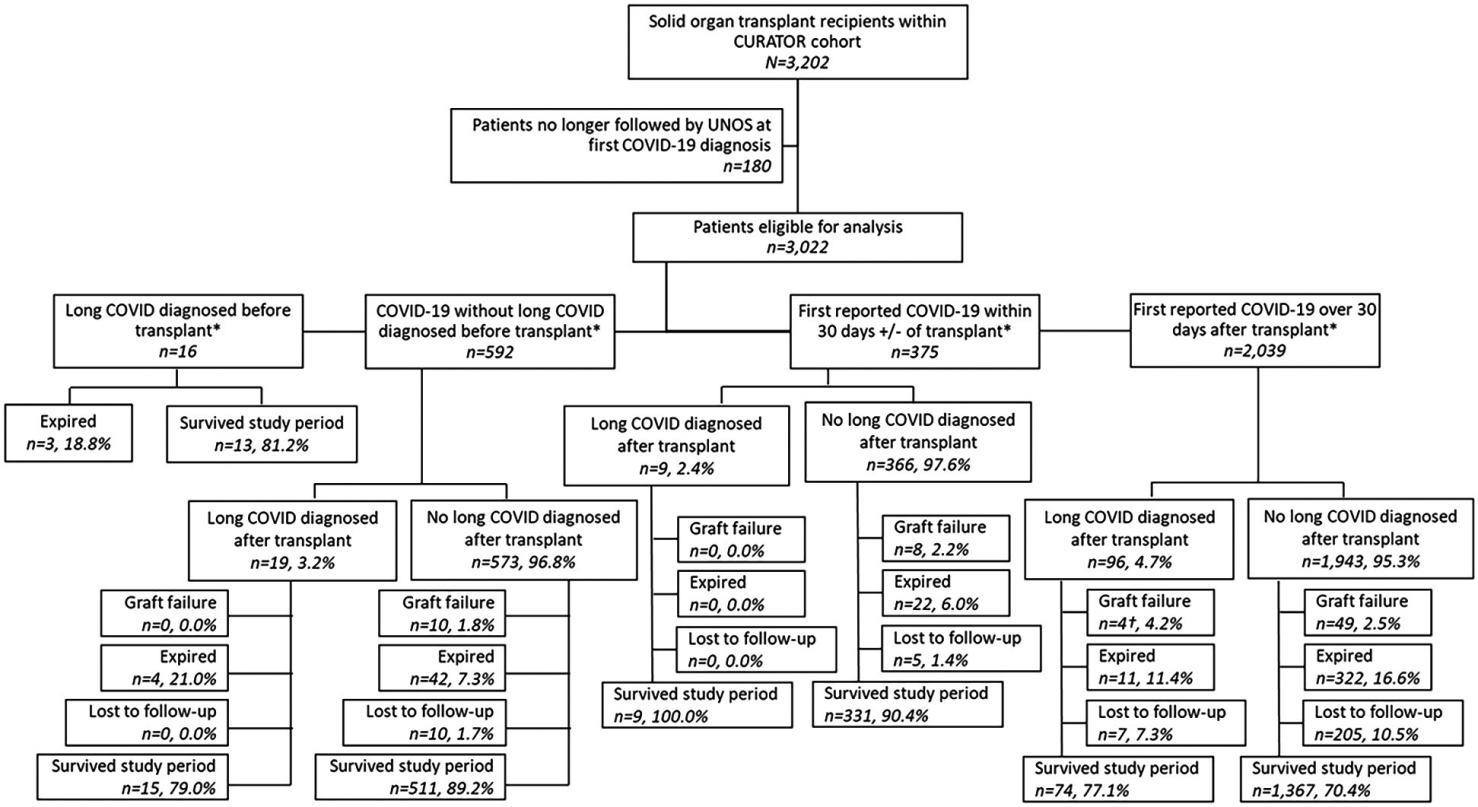
Study flow chart. *Transplant date recorded as date of most recent transplant. †Includes one patient diagnosed with long COVID after experiencing graft failure.

The temporal relationships between COVID-19 episodes, long COVID diagnoses, and transplants in this cohort were complex; to facilitate analyses, sub-cohorts were formed according to the temporal relationship between patients' first recorded COVID-19 episodes and their transplant date. The four sub-cohorts (Figure 1) are defined as follows: (1) patients who were diagnosed with long COVID before transplant; (2) patients whose first COVID-19 episode was recorded >30 days before transplant and who did not have a long COVID diagnosis recorded before transplant; (3) patients whose first COVID-19 episode was recorded within 30 days +/− of transplant; and (4) patients whose first COVID-19 episode was recorded > 30 days posttransplant. Patients were divided according to their long COVID status at the time of transplant to preserve the temporal relationship between the exposure (long COVID) and the outcome (graft failure or mortality). Further separation by COVID-19 status at the time of transplant was intended to segregate the competing risks of death due to acute COVID-19, acute transplant complications, and other causes in this highly comorbid population. Due to small cell values for the primary exposure, long COVID diagnoses, within each sub-cohort, additional statistical analyses such as multivariable logistic regression or Cox proportional hazard models incorporating time-varying exposure were not possible.

### 2.4 Post-COVID-19 transplant recipients

This sub-cohort consisted of patients whose first COVID-19 episode was recorded <30 days before transplant. For descriptive analyses, the observation window began at the date of transplant and ended at either 1 year (Outcome A) or 2 years (Outcome B). The primary exposure was long COVID diagnosed before the transplant date. Patients who were not followed for the entire observation window due to study end (15 December 2024) or loss to follow-up were excluded from descriptive analyses. For Kaplan–Meier analyses, time zero was the date of transplant, and composite events were recorded as the date of death or graft failure; patients were right-censored at study end or date of loss to follow-up.

### 2.5 COVID-19 within 30 days of transplant

This sub-cohort consists of patients whose first COVID-19 episode was recorded within 30 days plus or minus the transplant date. For descriptive analyses, the observation window began at the date of transplant and ended at either 90 days (Outcome A) or 1 year (Outcome B). The 90-day observation window was chosen to capture the risk of poor outcomes associated with acute COVID-19. Patients who received transplants within 90 days (Outcome A) or 1 year (Outcome B) of the study end date and those lost to follow-up were excluded from descriptive analyses. In this analysis of the acute COVID/transplant period, long COVID was not included. For Kaplan–Meier analyses, time zero was the date of transplant, and composite events were recorded as the date of death or graft failure; patients were right-censored at study end or date of loss to follow-up.

### 2.6 Post-SOT COVID-19

This sub-cohort consists of patients whose first COVID-19 episode was recorded <30 days after their transplant date. Two descriptive analyses were conducted in this sub-cohort, to differentiate between risk in the acute COVID-19 infection period (0–90 days post diagnosis; Outcome A) and risk in the post-acute period (6–18 months post period; Outcome B). For descriptive analyses of Outcome A, the observation window began at the date of the first recorded COVID-19 laboratory test positive and ended at either 90 days or at death/graft failure. Patients who were not followed for the entire observation window due to study end or loss to follow-up were excluded from descriptive analyses. In this analysis of the acute COVID/transplant period, long COVID was not included. For Kaplan–Meier analyses, time zero was the date of the first recorded COVID-19 laboratory test positive, and composite events were recorded as the date of death or graft failure; patients were right-censored at study end or date of loss to follow-up.

For descriptive analyses of Outcome B, the observation window began at 6 months (180 days) after the date of the first recorded COVID-19 laboratory test positive and ended at 18 months (545 days). The primary exposure was long COVID diagnosed in the 6 months following initial COVID-19 diagnosis. Patients who expired or experienced graft failure in the 6 months following COVID-19 diagnosis were excluded from descriptive analyses. Patients who were not followed for the entire observation window due to study end or loss to follow-up were excluded from descriptive analyses. Patients with a first recorded COVID-19 diagnosis date before 1 April 2021 were excluded, as they could not have received a long COVID diagnosis prior to the observation period.

For all descriptive analyses, differences across exposure groups were compared using Fisher's exact tests (categorical variables) or Kruskal–Wallis tests (continuous variables). All analyses were performed on Stata SE version 17.0 (StataCorp LLC, College Station, TX, USA). This retrospective registry-based study was approved by the Houston Methodist Institutional Review Board (PRO00025320) and granted a waiver of informed consent.

## 3 Results

### 3.1 Study population

**Figure 2:**
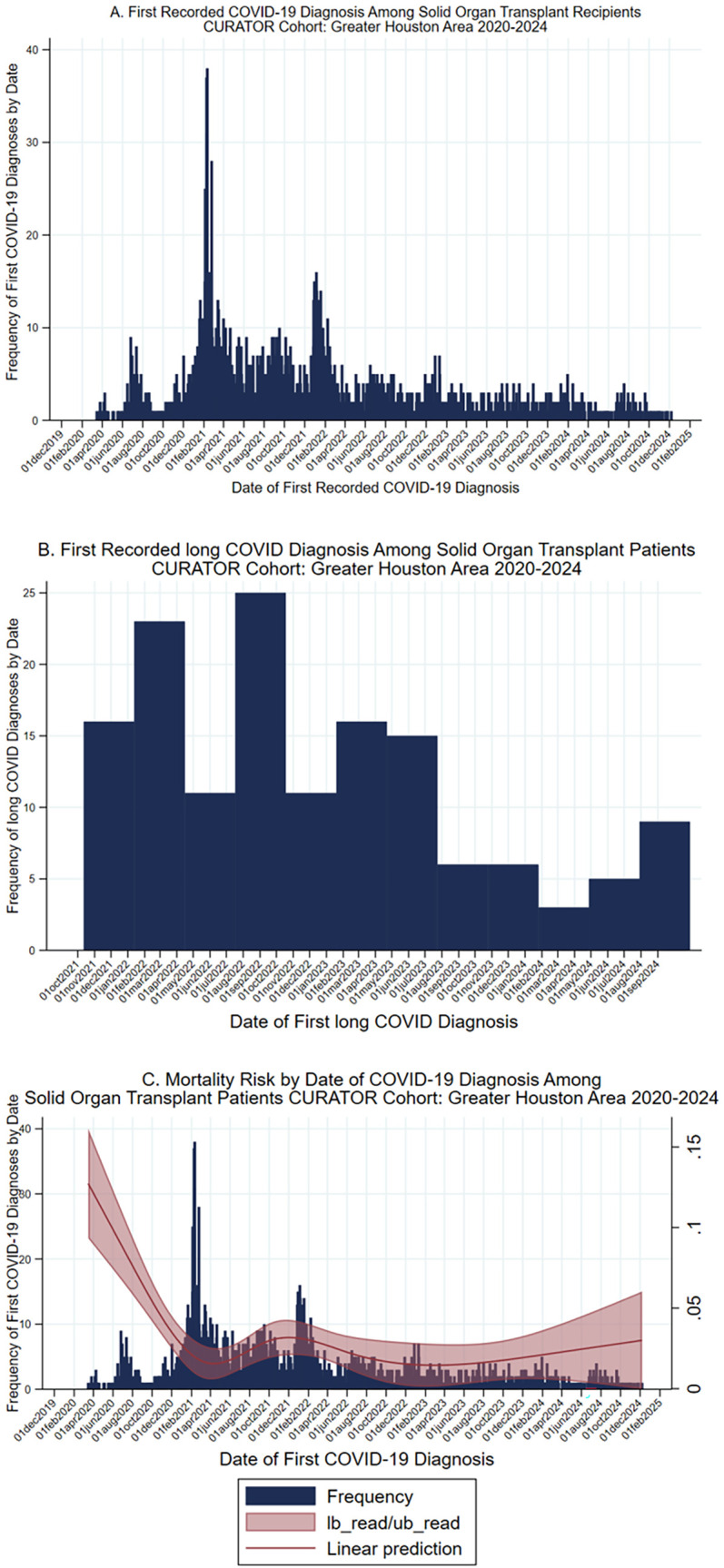
COVID-19, long COVID, and mortality risk over time. **(A)** Histogram, date of first recorded COVID-19 diagnosis among solid organ transplant recipients within the CURATOR cohort, 2020–2024. **(B)** First recorded long COVID diagnosis among solid organ transplant recipients within the CURATOR cohort, 2020–2024. Long COVID diagnosis determined by the presence of the ICD-10 code U09.9, which was instantiated on 1 October 2021. **(C)** Histogram, date of first recorded COVID-19 diagnosis among solid organ transplant recipients within the CURATOR cohort, 2020–2024, with natural spline of mortality risk in the 30 days post first COVID-19 diagnosis. CI, Confidence interval.

The total patient cohort (*n* = 3,202) with a history of SOT had been diagnosed with COVID-19 at the Houston Methodist Hospital system between 1 March 2020 and 15 December 2024 (Figure 1). Of these, 3,022 (94%, 3,022/3,202) were still being followed by UNOS at the time of their first COVID-19 diagnosis and were eligible for analysis. Within the study population, 608 patients were diagnosed with COVID-19, prior to their most recent transplant. Thirty-five (6%, 35/608) of these patients were further diagnosed with long COVID: 16 (46%, 16/35) had long COVID before their transplant, and 19 (54%, 19/35) were diagnosed after transplantation. An additional 375 patients' first recorded COVID-19 diagnoses occurred within 30 days plus or minus their transplant date, of whom 9 (2%, 9/375) were eventually diagnosed with long COVID. Finally, 2,039 patients were first diagnosed with COVID-19 >30 days after receiving SOT, of whom 96 (5%, 96/2,039) received a long COVID diagnosis. Histograms depict the first recorded COVID-19 diagnosis and the first recorded long COVID diagnosis by date (Figure 2).

In total, 443 (14%, 443/3,202) of the patients expired during the study period, 202 (6%, 202/3,202) were diagnosed with graft failure, and 272 (9%, 272/3,202) were lost to follow-up (Table 1). Of the 202 graft failure patients, 36 (18%, 36/202) eventually expired. The overall proportion of patients who died or experienced graft failure did not vary by long COVID status. Among patients who were not diagnosed with long COVID, 12.5% (218/1,738) died and 2.8% (49/1,738) experienced graft failure, while among patients who were diagnosed with long COVID, 11.7% (11/94) died and 4.3% (4/94) experienced graft failure. The most common cause of death was an infectious disease (*n* = 98, 22%), followed by cardiovascular disease (*n* = 47, 11%; Table 2). Notably, 83 (19%, 83/443) deaths were within 30 days of the patients' first recorded COVID-19 diagnosis.

**Table 1:** Demographics and clinical characteristics of solid organ transplant recipients diagnosed with COVID-19 by long COVID status.

Characteristics	Total	No long COVID	Long COVID	*p*-value
	*N* = 3,202	*N* = 3,056	*N* = 146	
Demographics at the first reported COVID-19 episode	*n* (%)	*n* (%)	*n* (%)	
Age at encounter (years), median (IQR)	59 (48–66)	59 (48–66)	62 (53–68)	0.022
Gender				0.39
Female	1,348 (42.1%)	1,292 (42.3%)	56 (38.4%)	
Male	1,854 (57.9%)	1,764 (57.7%)	90 (61.6%)	
Race/ethnicity				0.45
Non-Hispanic White	1,498 (46.8%)	1,432 (46.9%)	66 (45.2%)	
Non-Hispanic Black	691 (21.6%)	660 (21.6%)	31 (21.2%)	
Non-Hispanic Asian	188 (5.9%)	184 (6.0%)	4 (2.7%)	
Non-Hispanic Hawaiian/Pacific	6 (0.2%)	6 (0.2%)	0 (0.0%)	
Non-Hispanic Native American	9 (0.3%)	9 (0.3%)	0 (0.0%)	
Non-Hispanic other race	0 (0.0%)	0 (0.0%)	0 (0.0%)	
Hispanic or Latino	796 (24.9%)	752 (24.6%)	44 (30.1%)	
Unknown	14 (0.4%)	13 (0.4%)	1 (0.7%)	
Area deprivation index (state rank)				0.98
1–2 (Least deprivation)	864 (27.0%)	828 (27.1%)	36 (24.7%)	
3–4	804 (25.1%)	768 (25.1%)	36 (24.7%)	
5–6	619 (19.3%)	589 (19.3%)	30 (20.5%)	
7–8	531 (16.6%)	505 (16.5%)	26 (17.8%)	
9–10 (most deprivation)	329 (10.3%)	313 (10.2%)	16 (11.0%)	
Missing	55 (1.7%)	53 (1.7%)	2 (1.4%)	
Financial class at the first COVID-19 diagnosis				0.50
Private insurance	1,269 (39.6%)	1,213 (39.7%)	56 (38.4%)	
Medicare/Medicaid	1,831 (57.2%)	1,745 (57.1%)	86 (58.9%)	
Self-pay	38 (1.2%)	38 (1.2%)	0 (0.0%)	
Other	29 (0.9%)	27 (0.9%)	2 (1.4%)	
Missing	35 (1.1%)	33 (1.1%)	2 (1.4%)	
Clinical history (at the first COVID-19 diagnosis)				
Body mass index				0.56
BMI ≤ 18.5	44 (1.4%)	43 (1.4%)	1 (0.7%)	
BMI = 18.5–25	427 (13.3%)	409 (13.4%)	18 (12.3%)	
BMI = 25–30	489 (15.3%)	455 (14.9%)	34 (23.3%)	
BMI = 30–35	303 (9.5%)	287 (9.4%)	16 (11.0%)	
BMI = 35–40	100 (3.1%)	95 (3.1%)	5 (3.4%)	
BMI > 40	52 (1.6%)	50 (1.6%)	2 (1.4%)	
Missing	1,787 (55.8%)	1,717 (56.2%)	70 (47.9%)	
Charlson comorbidity index score				0.027
Score 1–2	73 (2.3%)	72 (2.4%)	1 (0.7%)	
Score 3–4	235 (7.3%)	231 (7.6%)	4 (2.7%)	
Score >4	2,894 (90.4%)	2,753 (90.1%)	141 (96.6%)	
Charlson comorbidity index components				
Asthma	488 (15.2%)	461 (15.1%)	27 (18.5%)	0.26
COPD	1,473 (46.0%)	1,366 (44.7%)	107 (73.3%)	<0.001
Tuberculosis	134 (4.2%)	126 (4.1%)	8 (5.5%)	0.42
Myocardial infarction	1,144 (35.7%)	1,067 (34.9%)	77 (52.7%)	<0.001
Congestive heart failure	1,742 (54.4%)	1,633 (53.4%)	109 (74.7%)	<0.001
Peripheral vascular disease	2,185 (68.2%)	2,064 (67.5%)	121 (82.9%)	<0.001
Cerebrovascular disease	1,643 (51.3%)	1,538 (50.3%)	105 (71.9%)	<0.001
Diabetes without complications	2,273 (71.0%)	2,153 (70.5%)	120 (82.2%)	0.002
Diabetes with complications	2,081 (65.0%)	1,972 (64.5%)	109 (74.7%)	0.012
Renal disease	3,005 (93.8%)	2,871 (93.9%)	134 (91.8%)	0.29
Peptic ulcer disease	400 (12.5%)	385 (12.6%)	15 (10.3%)	0.41
Mild liver disease	1,808 (56.5%)	1,720 (56.3%)	88 (60.3%)	0.34
Moderate to severe liver disease	820 (25.6%)	791 (25.9%)	29 (19.9%)	0.1
Dementia	98 (3.1%)	94 (3.1%)	4 (2.7%)	0.82
Hemiplegia	191 (6.0%)	179 (5.9%)	12 (8.2%)	0.24
Rheumatoid disease	391 (12.2%)	365 (11.9%)	26 (17.8%)	0.034
Cancer	832 (26.0%)	796 (26.0%)	36 (24.7%)	0.71
Metastatic cancer	404 (12.6%)	389 (12.7%)	15 (10.3%)	0.38
HIV/AIDS	44 (1.4%)	43 (1.4%)	1 (0.7%)	0.46
Transplant characteristics				
Transplanted organ				<0.001
Kidney	1,334 (41.7%)	1,299 (42.5%)	35 (24.0%)	
Liver	752 (23.5%)	732 (24.0%)	20 (13.7%)	
Heart	336 (10.5%)	311 (10.2%)	25 (17.1%)	
Lung	610 (19.1%)	547 (17.9%)	63 (43.2%)	
Heart–lung	29 (0.9%)	27 (0.9%)	2 (1.4%)	
Pancreas	9 (0.3%)	9 (0.3%)	0 (0.0%)	
Kidney–pancreas	131 (4.1%)	130 (4.3%)	1 (0.7%)	
Other (includes islet)	1 (0.0%)	1 (0.0%)	0 (0.0%)	
Donor type				0.004
Deceased donor (brain death)	2,268 (70.8%)	2,144 (70.2%)	124 (84.9%)	
Deceased donor (cardiac death)	191 (6.0%)	187 (6.1%)	4 (2.7%)	
Living donor	701 (21.9%)	685 (22.4%)	16 (11.0%)	
Unknown	42 (1.3%)	40 (1.3%)	2 (1.4%)	
Year of most recent transplant				0.54
Before 2018	1,182 (36.9%)	1,124 (36.8%)	58 (39.7%)	
2018	208 (6.5%)	197 (6.4%)	11 (7.5%)	
2019	262 (8.2%)	255 (8.3%)	7 (4.8%)	
2020	328 (10.2%)	313 (10.2%)	15 (10.3%)	
2021	374 (11.7%)	357 (11.7%)	17 (11.6%)	
2022	326 (10.2%)	308 (10.1%)	18 (12.3%)	
2023	306 (9.6%)	291 (9.5%)	15 (10.3%)	
2024	216 (6.7%)	211 (6.9%)	5 (3.4%)	
Time from transplant to COVID diagnosis				0.37
Diagnosed with COVID before transplant	787 (24.6%)	755 (24.7%)	32 (21.9%)	
COVID diagnosed at pretransplant evaluation	129 (4.0%)	122 (4.0%)	7 (4.8%)	
Diagnosed with COVID up to 30 days after transplant	73 (2.3%)	69 (2.3%)	4 (2.7%)	
Diagnosed with COVID 31–90 days after transplant	72 (2.2%)	72 (2.4%)	0 (0.0%)	
Diagnosed with COVID 91–180 days after transplant	97 (3.0%)	93 (3.0%)	4 (2.7%)	
Diagnosed with COVID over 180 days after transplant	2,044 (63.8%)	1,945 (63.6%)	99 (67.8%)	
Transplant outcome (first recorded event)^a^				0.81
Death	407 (12.7%)	389 (12.7%)	18 (12.3%)	
Graft failure	202 (6.3%)	195 (6.4%)	7 (4.8%)	
Loss to follow-up	272 (8.5%)	262 (8.6%)	10 (6.8%)	
Remained under observation	2,321 (72.5%)	2,210 (72.3%)	111 (76.0%)	
COVID-19 characteristics				
Date of the first recorded COVID-19 diagnosis (by local peak)				0.13
1 February 2020–15 September 2020	194 (6.1%)	183 (6.0%)	11 (7.5%)	
16 September 2020–20 June 2021	1,049 (32.8%)	1,011 (33.1%)	38 (26.0%)	
21 June 2021–20 November 2021	479 (15.0%)	456 (14.9%)	23 (15.8%)	
21 November 2021–1 April 2022	509 (15.9%)	481 (15.7%)	28 (19.2%)	
2 April 2022–15 October 2022	332 (10.4%)	309 (10.1%)	23 (15.8%)	
16 October 2022–1 June 2023	250 (7.8%)	239 (7.8%)	11 (7.5%)	
2 June 2023–15 November 2023	146 (4.6%)	142 (4.6%)	4 (2.7%)	
16 November 2023–1 May 2024	139 (4.3%)	137 (4.5%)	2 (1.4%)	
2 May 2024–15 December 2024	104 (3.2%)	98 (3.2%)	6 (4.1%)	
Status at the first recorded COVID-19 diagnostic encounter				
Hospitalized within 30 days	1,185 (37.0%)	1,115 (36.5%)	70 (47.9%)	0.005
Intensive care admission within 30 days	318 (9.9%)	295 (9.7%)	23 (15.8%)	0.016

Differences across exposure groups compared using Fisher's exact tests (categorical variables) or Kruskal–Wallis tests (continuous variables).

^a^ First recorded event displayed. Of the 202 patients who experienced graft failure, 36 expired during the study period, bringing the total observed deaths to 443.

**Table 2:** Characteristics of deceased solid organ transplant recipients diagnosed with COVID-19.

Characteristics	Total
	*N* = 443
	*n* (%)
Age at encounter (years), median (IQR)	64 (54–71)
Gender	
Female	178 (40.2%)
Male	265 (59.8%)
Body mass index	
BMI ≤ 18.5	13 (2.9%)
BMI = 18.5–25	94 (21.2%)
BMI = 25–30	87 (19.6%)
BMI = 30–35	38 (8.6%)
BMI = 35–40	19 (4.3%)
BMI > 40	8 (1.8%)
Missing	184 (41.5%)
Transplanted organ	
Kidney	116 (26.2%)
Liver	95 (21.4%)
Heart	58 (13.1%)
Lung	149 (33.6%)
Heart–lung	8 (1.8%)
Pancreas	1 (0.2%)
Kidney–pancreas	16 (3.6%)
Donor type	
Deceased donor (brain death)	376 (84.9%)
Deceased donor (cardiac death)	20 (4.5%)
Living donor	44 (9.9%)
Unknown	3 (0.7%)
UNOS primary cause of death	
Infectious disease	98 (22.1%)
Cardiovascular	47 (10.6%)
Cerebrovascular	5 (1.1%)
Pulmonary	41 (9.3%)
Malignancy	29 (6.5%)
Multiple organ system failure	25 (5.6%)
Graft failure	22 (5.0%)
Renal	15 (3.4%)
Other/unspecified	161 (36.3%)
Year of most recent transplant	
Before 2018	220 (49.7%)
2018	33 (7.4%)
2019	41 (9.3%)
2020	46 (10.4%)
2021	58 (13.1%)
2022	27 (6.1%)
2023	12 (2.7%)
2024	6 (1.4%)
Time between transplant and death	
Death within 12 months	37 (8.4%)
Death within 13–24 months	32 (7.2%)
Death within 24–48 months	43 (9.7%)
Death more than 48 months	331 (74.7%)
Time between COVID-19 diagnosis and death	
Death within 30 days of the first recorded COVID-19	83 (18.7%)
Time between long COVID diagnosis and death	
No long COVID	424 (95.7%)
Death within 90 days of long COVID diagnosis	3 (0.7%)
Death more than 90 days after long COVID diagnosis	16 (3.6%)
Date of last recorded COVID-19 diagnosis (by local peak)	
1 February 2020–15 September 2020	28 (6.3%)
16 September 2020–20 June 2021	88 (19.9%)
21 June 2021–20 November 2021	68 (15.3%)
21 November 2021–1 April 2022	106 (23.9%)
2 April 2022–15 October 2022	40 (9.0%)
16 October 2022–1 June 2023	61 (13.8%)
2 June 2023–15 November 2023	26 (5.9%)
16 November 2023–1 May 2024	17 (3.8%)
2 May 2024–15 December 2024	9 (2.0%)

Differences across exposure groups compared using Fisher's exact tests (categorical variables) or Kruskal–Wallis tests (continuous variables).

### 3.2 Long COVID

Overall, patients who were diagnosed with long COVID were older, had an increased comorbidity burden, and were more likely to be lung, heart, or heart–lung recipients compared with those who were not diagnosed with long COVID (Table 1). Patients with long COVID were additionally more likely to have chronic obstructive pulmonary disease, a history of myocardial infarction, congestive heart failure, peripheral vascular disease, cerebrovascular disease, diabetes, and rheumatoid disease than those without long COVID diagnoses. Patients who were hospitalized within 30 days of their first recorded COVID-19 episode were additionally more likely to receive a long COVID diagnosis. Of note, among the 16 patients who received transplants after being diagnosed with long COVID, 14 (88%, 14/16) were lung transplant recipients.

To explore factors associated with long COVID diagnosed after transplant, SOT recipients who survived at least 90 days following their first COVID-19 episode were evaluated (Table 3). A parallel analysis, presented in Supplementary Table S1, is limited to patients diagnosed with COVID-19 between October 2021 and August 2024, since long COVID was not recognized as an ICD-10 diagnosis code until 1 October 2021 (27). The clinical risk factors associated with long COVID in this population of COVID-19 survivors were similar to the overall cohort (Table 1), with notable exceptions: liver patients were less likely to be diagnosed with long COVID, and rheumatoid disease was not significantly associated with long COVID diagnosis.

**Table 3:** Ninety-day COVID survivor solid organ transplant recipients by long COVID status, 2020–2024.

Characteristics	Total	No long COVID	Long COVID	*p*-value
	*N* = 1,832	*N* = 1,738	*N* = 146	
Demographics at the first reported COVID-19 episode	*n* (%)	*n* (%)	*n* (%)	
Age at encounter (years), median (IQR)	60 (49–67)	59 (49–67)	62 (55–68)	0.1
Gender				0.83
Female	767 (41.9%)	729 (41.9%)	38 (40.4%)	
Male	1,065 (58.1%)	1,009 (58.1%)	56 (59.6%)	
Race/ethnicity				0.48
Non-Hispanic White	932 (50.9%)	886 (51.0%)	46 (48.9%)	
Non-Hispanic Black	372 (20.3%)	351 (20.2%)	21 (22.3%)	
Non-Hispanic Asian	106 (5.8%)	104 (6.0%)	2 (2.1%)	
Non-Hispanic Hawaiian/Pacific	5 (0.3%)	5 (0.3%)	0 (0.0%)	
Non-Hispanic Native American	5 (0.3%)	5 (0.3%)	0 (0.0%)	
Non-Hispanic other race	0 (0.0%)	0 (0.0%)	0 (0.0%)	
Hispanic or Latino	404 (22.1%)	380 (21.9%)	24 (25.5%)	
Unknown	8 (0.4%)	7 (0.4%)	1 (1.1%)	
Area deprivation index (state rank)				0.97
1–2 (least deprivation)	504 (27.5%)	478 (27.5%)	26 (27.7%)	
3–4	459 (25.1%)	436 (25.1%)	23 (24.5%)	
5–6	354 (19.3%)	334 (19.2%)	20 (21.3%)	
7–8	297 (16.2%)	281 (16.2%)	16 (17.0%)	
9–10 (most deprivation)	193 (10.5%)	184 (10.6%)	9 (9.6%)	
Missing	25 (1.4%)	25 (1.4%)	0 (0.0%)	
Financial class at the first COVID-19 diagnosis				0.74
Private insurance	671 (36.6%)	640 (36.8%)	31 (33.0%)	
Medicare/Medicaid	1,108 (60.5%)	1,046 (60.2%)	62 (66.0%)	
Self-pay	15 (0.8%)	15 (0.9%)	0 (0.0%)	
Other	14 (0.8%)	14 (0.8%)	0 (0.0%)	
Missing	24 (1.3%)	23 (1.3%)	1 (1.1%)	
Clinical history (at the first COVID-19 diagnosis)				
Body mass index				0.97
BMI ≤ 18.5	25 (1.4%)	24 (1.4%)	1 (1.1%)	
BMI = 18.5–25	225 (12.3%)	214 (12.3%)	11 (11.7%)	
BMI = 25–30	262 (14.3%)	243 (14.0%)	19 (20.2%)	
BMI = 30–35	172 (9.4%)	163 (9.4%)	9 (9.6%)	
BMI = 35–40	49 (2.7%)	46 (2.6%)	3 (3.2%)	
BMI > 40	26 (1.4%)	24 (1.4%)	2 (2.1%)	
Missing	1,073 (58.6%)	1,024 (58.9%)	49 (52.1%)	
Charlson comorbidity index score				0.88
Score 1–2	37 (2.0%)	37 (2.1%)	0 (0.0%)	
Score 3–4	123 (6.7%)	119 (6.8%)	4 (4.3%)	
Score > 4	1,672 (91.3%)	1,582 (91.0%)	90 (95.7%)	
Charlson comorbidity index components				
Asthma	268 (14.6%)	251 (14.4%)	17 (18.1%)	0.33
COPD	870 (47.5%)	803 (46.2%)	67 (71.3%)	<0.001
Tuberculosis	84 (4.6%)	79 (4.5%)	5 (5.3%)	0.73
Myocardial infarction	685 (37.4%)	638 (36.7%)	47 (50.0%)	0.009
Congestive heart failure	1,003 (54.7%)	937 (53.9%)	66 (70.2%)	0.002
Peripheral vascular disease	1,299 (70.9%)	1,218 (70.1%)	81 (86.2%)	<0.001
Cerebrovascular disease	965 (52.7%)	900 (51.8%)	65 (69.1%)	0.001
Diabetes without complications	1,337 (73.0%)	1,260 (72.5%)	77 (81.9%)	0.045
Diabetes with complications	1,227 (67.0%)	1,157 (66.6%)	70 (74.5%)	0.11
Renal disease	1,726 (94.2%)	1,636 (94.1%)	90 (95.7%)	0.51
Peptic ulcer disease	227 (12.4%)	216 (12.4%)	11 (11.7%)	0.84
Mild liver disease	1,120 (61.1%)	1,060 (61.0%)	60 (63.8%)	0.58
Moderate to severe liver disease	518 (28.3%)	501 (28.8%)	17 (18.1%)	0.024
Dementia	64 (3.5%)	61 (3.5%)	3 (3.2%)	0.87
Hemiplegia	118 (6.4%)	109 (6.3%)	9 (9.6%)	0.2
Rheumatoid disease	227 (12.4%)	210 (12.1%)	17 (18.1%)	0.085
Cancer	562 (30.7%)	538 (31.0%)	24 (25.5%)	0.27
Metastatic cancer	271 (14.8%)	258 (14.8%)	13 (13.8%)	0.79
HIV/AIDS	23 (1.3%)	23 (1.3%)	0 (0.0%)	0.26
Transplant characteristics				
Transplanted organ				<0.001
Kidney	584 (31.9%)	561 (32.3%)	23 (24.5%)	
Liver	508 (27.7%)	495 (28.5%)	13 (13.8%)	
Heart	248 (13.5%)	226 (13.0%)	22 (23.4%)	
Lung	359 (19.6%)	325 (18.7%)	34 (36.2%)	
Heart–lung	20 (1.1%)	18 (1.0%)	2 (2.1%)	
Pancreas	6 (0.3%)	6 (0.3%)	0 (0.0%)	
Kidney–pancreas	106 (5.8%)	106 (6.1%)	0 (0.0%)	
Other (includes islet)	1 (0.1%)	1 (0.1%)	0 (0.0%)	
Donor type				0.23
Deceased donor (brain death)	1,453 (79.3%)	1,373 (79.0%)	80 (85.1%)	
Deceased donor (cardiac death)	89 (4.9%)	87 (5.0%)	2 (2.1%)	
Living donor	264 (14.4%)	254 (14.6%)	10 (10.6%)	
Unknown	26 (1.4%)	24 (1.4%)	2 (2.1%)	
Year of most recent transplant				0.56
Before 2018	798 (43.6%)	751 (43.2%)	47 (50.0%)	
2018	188 (10.3%)	178 (10.2%)	10 (10.6%)	
2019	237 (12.9%)	231 (13.3%)	6 (6.4%)	
2020	293 (16.0%)	280 (16.1%)	13 (13.8%)	
2021	156 (8.5%)	147 (8.5%)	9 (9.6%)	
2022	90 (4.9%)	84 (4.8%)	6 (6.4%)	
2023	56 (3.1%)	53 (3.0%)	3 (3.2%)	
2024	14 (0.8%)	14 (0.8%)	0 (0.0%)	
Transplant outcome (first recorded event)				0.47
Death	229 (12.5%)	218 (12.5%)	11 (11.7%)	
Graft failure	53 (2.9%)	49 (2.8%)	4 (4.3%)	
Loss to follow-up	45 (2.5%)	44 (2.5%)	1 (1.1%)	
Remained under observation	1,505 (82.2%)	1,427 (82.1%)	78 (83.0%)	
COVID-19 characteristics				
Date of the first recorded COVID-19 diagnosis (by local peak)				0.68
1 February 2020–15 September 2020	93 (5.1%)	87 (5.0%)	6 (6.4%)	
16 September 2020–20 June 2021	684 (37.3%)	653 (37.6%)	31 (33.0%)	
21 June 2021–20 November 2021	304 (16.6%)	287 (16.5%)	17 (18.1%)	
21 November 2021–1 April 2022	312 (17.0%)	297 (17.1%)	15 (16.0%)	
2 April 2022–15 October 2022	162 (8.8%)	150 (8.6%)	12 (12.8%)	
16 October 2022–1 June 2023	101 (5.5%)	94 (5.4%)	7 (7.4%)	
2 June 2023–15 November 2023	51 (2.8%)	49 (2.8%)	2 (2.1%)	
16 November 2023–1 May 2024	73 (4.0%)	72 (4.1%)	1 (1.1%)	
2 May 2024–15 December 2024	52 (2.8%)	49 (2.8%)	3 (3.2%)	
Status at the first recorded COVID-19 diagnostic encounter				
Hospitalized within 30 days	609 (33.2%)	569 (32.7%)	40 (42.6%)	0.049
Intensive care admission within 30 days	108 (5.9%)	97 (5.6%)	11 (11.7%)	0.014

Differences across exposure groups compared using Fisher's exact tests (categorical variables) or Kruskal–Wallis tests (continuous variables).

Includes SOT recipients who survived at least 90 days following their first recorded COVID-19 laboratory test positive. Exposures: demographic and clinical variables abstracted from EMR at the first COVID-19 diagnostic encounter. Outcome: long COVID diagnosis determined by the presence of U09.9 ICD-10 code. Patients with a first recorded COVID-19 laboratory test positive before their most recent transplant were excluded from this analysis.

### 3.3 Post-COVID-19 transplant recipients

Table 4 compares the composite outcome of death or graft failure at 1 or 2 years posttransplant among patients whose first COVID-19 episode was recorded >30 days before transplant (1 year, *N* = 421, 25 events; 2 years, *N* = 261, 33 events). While being diagnosed with long COVID before transplant was not associated with the composite outcome (1-year outcomes, *p* = 0.58; 2-year outcomes, *p* = 0.44), a history of myocardial infarction, congestive heart failure, and peptic ulcer disease were associated with 1-year mortality/graft failure while rheumatoid disease and metastatic cancer were associated with 2-year mortality/graft failure.

**Table 4:** One- and 2-year death/graft failure among post-COVID-19 solid organ transplant recipients.

Characteristics	(A) Death/graft failure: 1 year posttransplant				(B) Death/graft failure: 2 years posttransplant			
	Total *N* = 421	No events *n* = 396	Events *n* = 25	*p*-value	Total *N* = 261	No events *n* = 228	Events *n* = 33	*p*-value
Demographics at the first reported COVID-19 episode	*n* (%)	*n* (%)	*n* (%)		*n* (%)	*n* (%)	*n* (%)	
Age at encounter (years), median (IQR)	56 (46–63)	55 (46–63)	60 (46–63)	0.28	56 (45–63)	56 (46–62.5)	54 (43–63)	0.85
Gender				0.68				0.99
Female	177 (42.0%)	168 (42.4%)	9 (36.0%)		99 (37.9%)	87 (38.2%)	12 (36.4%)	
Male	244 (58.0%)	228 (57.6%)	16 (64.0%)		162 (62.1%)	141 (61.8%)	21 (63.6%)	
Race/ethnicity				0.098				0.46
Non-Hispanic White	185 (43.9%)	172 (43.4%)	13 (52.0%)		114 (43.7%)	99 (43.4%)	15 (45.5%)	
Non-Hispanic Black	91 (21.6%)	82 (20.7%)	9 (36.0%)		59 (22.6%)	48 (21.1%)	11 (33.3%)	
Non-Hispanic Asian	15 (3.6%)	14 (3.5%)	1 (4.0%)		10 (3.8%)	9 (3.9%)	1 (3.0%)	
Non-Hispanic Hawaiian/Pacific	1 (0.2%)	1 (0.3%)	0 (0.0%)		0 (0.0%)	0 (0.0%)	0 (0.0%)	
Non-Hispanic Native American	1 (0.2%)	1 (0.3%)	0 (0.0%)		1 (0.4%)	1 (0.4%)	0 (0.0%)	
Non-Hispanic other race	0 (0.0%)	0 (0.0%)	0 (0.0%)		0 (0.0%)	0 (0.0%)	0 (0.0%)	
Hispanic or Latino	127 (30.2%)	125 (31.6%)	2 (8.0%)		76 (29.1%)	70 (30.7%)	6 (18.2%)	
Unknown	1 (0.2%)	1 (0.3%)	0 (0.0%)		1 (0.4%)	1 (0.4%)	0 (0.0%)	
Area deprivation index (state rank)				0.48				0.77
1–2 (least deprivation)	108 (25.7%)	103 (26.0%)	5 (20.0%)		69 (26.4%)	59 (25.9%)	10 (30.3%)	
3–4	110 (26.1%)	103 (26.0%)	7 (28.0%)		67 (25.7%)	60 (26.3%)	7 (21.2%)	
5–6	85 (20.2%)	80 (20.2%)	5 (20.0%)		53 (20.3%)	47 (20.6%)	6 (18.2%)	
7–8	79 (18.8%)	72 (18.2%)	7 (28.0%)		47 (18.0%)	41 (18.0%)	6 (18.2%)	
9–10 (most deprivation)	30 (7.1%)	30 (7.6%)	0 (0.0%)		18 (6.9%)	16 (7.0%)	2 (6.1%)	
Missing	9 (2.1%)	8 (2.0%)	1 (4.0%)		7 (2.7%)	5 (2.2%)	2 (6.1%)	
Financial class at the first COVID-19 diagnosis				0.76				0.82
Private insurance	227 (53.9%)	211 (53.3%)	16 (64.0%)		134 (51.3%)	117 (51.3%)	17 (51.5%)	
Medicare/Medicaid	180 (42.8%)	171 (43.2%)	9 (36.0%)		119 (45.6%)	104 (45.6%)	15 (45.5%)	
Self-pay	9 (2.1%)	9 (2.3%)	0 (0.0%)		5 (1.9%)	4 (1.8%)	1 (3.0%)	
Other	5 (1.2%)	5 (1.3%)	0 (0.0%)		3 (1.1%)	3 (1.3%)	0 (0.0%)	
Clinical history (at the first COVID-19 diagnosis)								
Body mass index				0.19				0.91
BMI ≤ 18.5	5 (1.2%)	5 (1.3%)	0 (0.0%)		5 (1.9%)	5 (2.2%)	0 (0.0%)	
BMI = 18.5–25	46 (10.9%)	45 (11.4%)	1 (4.0%)		21 (8.0%)	18 (7.9%)	3 (9.1%)	
BMI = 25–30	65 (15.4%)	62 (15.7%)	3 (12.0%)		35 (13.4%)	31 (13.6%)	4 (12.1%)	
BMI = 30–35	34 (8.1%)	30 (7.6%)	4 (16.0%)		25 (9.6%)	22 (9.6%)	3 (9.1%)	
BMI = 35–40	14 (3.3%)	13 (3.3%)	1 (4.0%)		10 (3.8%)	9 (3.9%)	1 (3.0%)	
BMI > 40	10 (2.4%)	8 (2.0%)	2 (8.0%)		4 (1.5%)	3 (1.3%)	1 (3.0%)	
Missing	247 (58.7%)	233 (58.8%)	14 (56.0%)		161 (61.7%)	140 (61.4%)	21 (63.6%)	
Charlson comorbidity index components								
Asthma	74 (17.6%)	71 (17.9%)	3 (12.0%)	0.45	45 (17.2%)	41 (18.0%)	4 (12.1%)	0.4
COPD	218 (51.8%)	206 (52.0%)	12 (48.0%)	0.7	130 (49.8%)	111 (48.7%)	19 (57.6%)	0.34
Tuberculosis	17 (4.0%)	17 (4.3%)	0 (0.0%)	0.29	9 (3.4%)	7 (3.1%)	2 (6.1%)	0.38
Myocardial infarction	160 (38.0%)	144 (36.4%)	16 (64.0%)	0.006	97 (37.2%)	80 (35.1%)	17 (51.5%)	0.068
Congestive heart failure	256 (60.8%)	235 (59.3%)	21 (84.0%)	0.014	157 (60.2%)	133 (58.3%)	24 (72.7%)	0.11
Peripheral vascular disease	308 (73.2%)	287 (72.5%)	21 (84.0%)	0.21	191 (73.2%)	166 (72.8%)	25 (75.8%)	0.72
Cerebrovascular disease	262 (62.2%)	245 (61.9%)	17 (68.0%)	0.54	160 (61.3%)	136 (59.6%)	24 (72.7%)	0.15
Diabetes without complications	50 (11.9%)	48 (12.1%)	2 (8.0%)	0.54	30 (11.5%)	24 (10.5%)	6 (18.2%)	0.20
Diabetes with complications	285 (67.7%)	266 (67.2%)	19 (76.0%)	0.36	173 (66.3%)	150 (65.8%)	23 (69.7%)	0.66
Renal disease	392 (93.1%)	367 (92.7%)	25 (100.0%)	0.16	249 (95.4%)	218 (95.6%)	31 (93.9%)	0.67
Peptic ulcer disease	58 (13.8%)	51 (12.9%)	7 (28.0%)	0.033	38 (14.6%)	32 (14.0%)	6 (18.2%)	0.53
Mild liver disease	130 (30.9%)	122 (30.8%)	8 (32.0%)	0.9	83 (31.8%)	74 (32.5%)	9 (27.3%)	0.55
Moderate to severe liver disease	145 (34.4%)	134 (33.8%)	11 (44.0%)	0.3	87 (33.3%)	72 (31.6%)	15 (45.5%)	0.11
Dementia	8 (1.9%)	8 (2.0%)	0 (0.0%)	0.47	4 (1.5%)	4 (1.8%)	0 (0.0%)	0.44
Hemiplegia	22 (5.2%)	21 (5.3%)	1 (4.0%)	0.78	13 (5.0%)	11 (4.8%)	2 (6.1%)	0.76
Rheumatoid disease	48 (11.4%)	45 (11.4%)	3 (12.0%)	0.92	30 (11.5%)	22 (9.6%)	8 (24.2%)	0.014
Cancer	98 (23.3%)	90 (22.7%)	8 (32.0%)	0.29	70 (26.8%)	59 (25.9%)	11 (33.3%)	0.37
Metastatic cancer	37 (8.8%)	33 (8.3%)	4 (16.0%)	0.19	29 (11.1%)	20 (8.8%)	9 (27.3%)	0.002
HIV/AIDS	10 (2.4%)	9 (2.3%)	1 (4.0%)	0.58	7 (2.7%)	6 (2.6%)	1 (3.0%)	0.89
Transplant characteristics								
Transplanted organ				0.23				0.3
Kidney	130 (30.9%)	125 (31.6%)	5 (20.0%)		86 (33.0%)	80 (35.1%)	6 (18.2%)	
Liver	112 (26.6%)	104 (26.3%)	8 (32.0%)		73 (28.0%)	62 (27.2%)	11 (33.3%)	
Heart	45 (10.7%)	39 (9.8%)	6 (24.0%)		32 (12.3%)	26 (11.4%)	6 (18.2%)	
Lung	119 (28.3%)	113 (28.5%)	6 (24.0%)		58 (22.2%)	49 (21.5%)	9 (27.3%)	
Heart–lung	6 (1.4%)	6 (1.5%)	0 (0.0%)		4 (1.5%)	3 (1.3%)	1 (3.0%)	
Pancreas	0 (0.0%)	0 (0.0%)	0 (0.0%)		0 (0.0%)	0 (0.0%)	0 (0.0%)	
Kidney–pancreas	9 (2.1%)	9 (2.3%)	0 (0.0%)		8 (3.1%)	8 (3.5%)	0 (0.0%)	
Other (includes islet)	0 (0.0%)	0 (0.0%)	0 (0.0%)		0 (0.0%)	0 (0.0%)	0 (0.0%)	
Donor type				0.35				0.058
Deceased donor (brain death)	301 (71.5%)	280 (70.7%)	21 (84.0%)		183 (70.1%)	154 (67.5%)	29 (87.9%)	
Deceased donor (cardiac death)	39 (9.3%)	38 (9.6%)	1 (4.0%)		21 (8.0%)	20 (8.8%)	1 (3.0%)	
Living donor	81 (19.2%)	78 (19.7%)	3 (12.0%)		57 (21.8%)	54 (23.7%)	3 (9.1%)	
COVID-19 characteristics								
Date of the first recorded COVID-19 diagnosis (by local peak)				0.093				0.75
1 February 2020–15 September 2020	32 (7.6%)	28 (7.1%)	4 (16.0%)		24 (9.2%)	20 (8.8%)	4 (12.1%)	
16 September 2020–20 June 2021	160 (38.0%)	146 (36.9%)	14 (56.0%)		138 (52.9%)	118 (51.8%)	20 (60.6%)	
21 June 2021–20 November 2021	44 (10.5%)	43 (10.9%)	1 (4.0%)		32 (12.3%)	29 (12.7%)	3 (9.1%)	
21 November 2021–1 April 2022	70 (16.6%)	67 (16.9%)	3 (12.0%)		46 (17.6%)	42 (18.4%)	4 (12.1%)	
2 April 2022–15 October 2022	59 (14.0%)	59 (14.9%)	0 (0.0%)		21 (8.0%)	19 (8.3%)	2 (6.1%)	
16 October 2022–1 June 2023	42 (10.0%)	39 (9.8%)	3 (12.0%)		0 (0.0%)	0 (0.0%)	0 (0.0%)	
2 June 2023–15 November 2023	14 (3.3%)	14 (3.5%)	0 (0.0%)		0 (0.0%)	0 (0.0%)	0 (0.0%)	
16 November 2023–1 May 2024	0 (0.0%)	0 (0.0%)	0 (0.0%)		0 (0.0%)	0 (0.0%)	0 (0.0%)	
Status at the first recorded COVID-19 diagnostic encounter								
Hospitalized within 30 days	150 (35.6%)	140 (35.4%)	10 (40.0%)	0.64	92 (35.2%)	82 (36.0%)	10 (30.3%)	0.52
Intensive care admission within 30 days	48 (11.4%)	46 (11.6%)	2 (8.0%)	0.58	33 (12.6%)	29 (12.7%)	4 (12.1%)	0.92
Long COVID diagnosis reported before transplant date	10 (2.4%)	9 (2.3%)	1 (4.0%)	0.58	4 (1.5%)	4 (1.8%)	0 (0.0%)	0.44

Differences across exposure groups compared using Fisher's exact tests (categorical variables) or Kruskal–Wallis tests (continuous variables).

Includes patients with a COVID-19 laboratory test positive recorded >30 days before transplant. Exposures: demographic and clinical variables abstracted from EMR at the first COVID-19 diagnostic encounter. Outcome: composite all-cause mortality or graft failure within 1 year or 2 years of transplant date. Patients who received transplants within 1 year (Outcome A) or 2 years (Outcome B) of the study end date and those lost to follow-up were excluded.

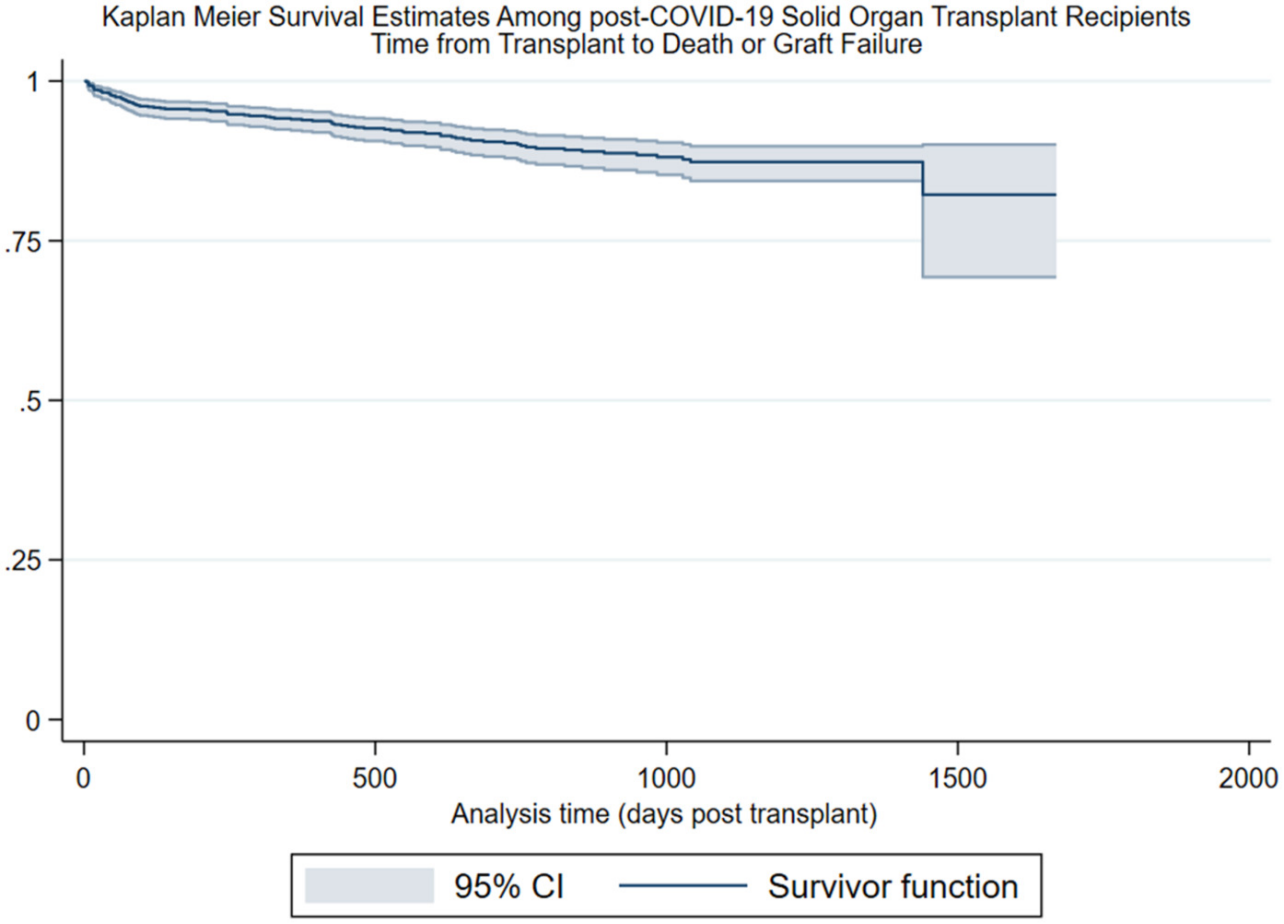

Legend: Kaplan–Meier analyses, the *y*-axis represents survivorship, the *x*-axis represents analysis, time zero was the date of transplant, and composite events were recorded as the date of death or graft failure; patients were right-censored at study end or date of loss to follow-up. CI, confidence interval.

Survival function at 365 days: 0.94, CI: 0.91–0.95.

Survival function at 730 days: 0.88, CI: 0.85–0.91.

### 3.4 COVID-19 within 30 days of transplant

Table 5 compares the composite outcome of death or graft failure at 90 days or 1 year posttransplant among patients whose first COVID-19 episode was recorded within 30 days plus or minus the transplant date (90 days, *N* = 369, 14 events; 1 year, *N* = 326, 20 events). Diabetes was the only risk factor associated with the 90-day composite outcome, while myocardial infarction history and being a liver patient were associated with 1-year death/graft failure.

**Table 5:** Ninety-day and 1-year death/graft failure among solid organ transplant recipients diagnosed with COVID-19 within 30 days of transplant.

Characteristics	(A) Death/graft failure: 90 days posttransplant				(B) Death/graft failure: 1-year posttransplant			
	Total *N* = 369	No events, *n* = 355	Events *n* = 14	*p*-value	Total *N* = 326	No events, *n* = 306	Events *n* = 20	*p*-value
Demographics at the first reported COVID-19 episode	*n* (%)	*n* (%)	*n* (%)		*n* (%)	*n* (%)	*n* (%)	
Age at encounter (years), median (IQR)	54 (44–63)	53 (44–63)	57 (49–65)	0.38	53.5 (44–63)	53 (44–63)	58 (51–67.5)	0.072
Gender				0.99				0.48
Female	149 (40.4%)	143 (40.3%)	6 (42.9%)		132 (40.5%)	122 (39.9%)	10 (50.0%)	
Male	220 (59.6%)	212 (59.7%)	8 (57.1%)		194 (59.5%)	184 (60.1%)	10 (50.0%)	
Race/ethnicity				0.57				0.6
Non-Hispanic White	141 (38.2%)	136 (38.3%)	5 (35.7%)		122 (37.4%)	114 (37.3%)	8 (40.0%)	
Non-Hispanic Black	88 (23.8%)	83 (23.4%)	5 (35.7%)		83 (25.5%)	76 (24.8%)	7 (35.0%)	
Non-Hispanic Asian	39 (10.6%)	37 (10.4%)	2 (14.3%)		32 (9.8%)	30 (9.8%)	2 (10.0%)	
Non-Hispanic Hawaiian/Pacific	0 (0.0%)	0 (0.0%)	0 (0.0%)		0 (0.0%)	0 (0.0%)	0 (0.0%)	
Non-Hispanic Native American	0 (0.0%)	0 (0.0%)	0 (0.0%)		0 (0.0%)	0 (0.0%)	0 (0.0%)	
Non-Hispanic other race	0 (0.0%)	0 (0.0%)	0 (0.0%)		0 (0.0%)	0 (0.0%)	0 (0.0%)	
Hispanic or Latino	100 (27.1%)	98 (27.6%)	2 (14.3%)		88 (27.0%)	85 (27.8%)	3 (15.0%)	
Unknown	1 (0.3%)	1 (0.3%)	0 (0.0%)		1 (0.3%)	1 (0.3%)	0 (0.0%)	
Area deprivation index (state rank)				0.81				0.37
1–2 (Least deprivation)	113 (30.6%)	108 (30.4%)	5 (35.7%)		98 (30.1%)	90 (29.4%)	8 (40.0%)	
3–4	91 (24.7%)	87 (24.5%)	4 (28.6%)		81 (24.8%)	76 (24.8%)	5 (25.0%)	
5–6	65 (17.6%)	64 (18.0%)	1 (7.1%)		56 (17.2%)	55 (18.0%)	1 (5.0%)	
7–8	52 (14.1%)	49 (13.8%)	3 (21.4%)		47 (14.4%)	42 (13.7%)	5 (25.0%)	
9–10 (most deprivation)	44 (11.9%)	43 (12.1%)	1 (7.1%)		41 (12.6%)	40 (13.1%)	1 (5.0%)	
Missing	4 (1.1%)	4 (1.1%)	0 (0.0%)		3 (0.9%)	3 (1.0%)	0 (0.0%)	
Financial class at the first COVID-19 diagnosis				0.23				0.21
Private insurance	164 (44.4%)	161 (45.4%)	3 (21.4%)		142 (43.6%)	137 (44.8%)	5 (25.0%)	
Medicare/Medicaid	201 (54.5%)	190 (53.5%)	11 (78.6%)		182 (55.8%)	167 (54.6%)	15 (75.0%)	
Self-pay	1 (0.3%)	1 (0.3%)	0 (0.0%)		1 (0.3%)	1 (0.3%)	0 (0.0%)	
Other	3 (0.8%)	3 (0.8%)	0 (0.0%)		1 (0.3%)	1 (0.3%)	0 (0.0%)	
Clinical history (at the first COVID-19 diagnosis)								
Body mass index				0.59				0.68
BMI ≤ 18.5	6 (1.6%)	6 (1.7%)	0 (0.0%)		5 (1.5%)	5 (1.6%)	0 (0.0%)	
BMI = 18.5–25	38 (10.3%)	35 (9.9%)	3 (21.4%)		34 (10.4%)	30 (9.8%)	4 (20.0%)	
BMI = 25–30	56 (15.2%)	55 (15.5%)	1 (7.1%)		51 (15.6%)	47 (15.4%)	4 (20.0%)	
BMI = 30–35	40 (10.8%)	39 (11.0%)	1 (7.1%)		37 (11.3%)	36 (11.8%)	1 (5.0%)	
BMI = 35–40	5 (1.4%)	5 (1.4%)	0 (0.0%)		5 (1.5%)	5 (1.6%)	0 (0.0%)	
BMI > 40	3 (0.8%)	3 (0.8%)	0 (0.0%)		3 (0.9%)	3 (1.0%)	0 (0.0%)	
Missing	221 (59.9%)	212 (59.7%)	9 (64.3%)		191 (58.6%)	180 (58.8%)	11 (55.0%)	
Charlson comorbidity index components								
Asthma	53 (14.4%)	51 (14.4%)	2 (14.3%)	0.99	49 (15.0%)	47 (15.4%)	2 (10.0%)	0.52
COPD	100 (27.1%)	94 (26.5%)	6 (42.9%)	0.18	90 (27.6%)	81 (26.5%)	9 (45.0%)	0.073
Tuberculosis	7 (1.9%)	6 (1.7%)	1 (7.1%)	0.14	6 (1.8%)	5 (1.6%)	1 (5.0%)	0.28
Myocardial infarction	87 (23.6%)	82 (23.1%)	5 (35.7%)	0.28	79 (24.2%)	70 (22.9%)	9 (45.0%)	0.025
Congestive heart failure	143 (38.8%)	137 (38.6%)	6 (42.9%)	0.75	130 (39.9%)	119 (38.9%)	11 (55.0%)	0.15
Peripheral vascular disease	222 (60.2%)	216 (60.8%)	6 (42.9%)	0.18	194 (59.5%)	183 (59.8%)	11 (55.0%)	0.67
Cerebrovascular disease	140 (37.9%)	134 (37.7%)	6 (42.9%)	0.7	124 (38.0%)	114 (37.3%)	10 (50.0%)	0.26
Diabetes without complications	34 (9.2%)	34 (9.6%)	0 (0.0%)	0.22	27 (8.3%)	26 (8.5%)	1 (5.0%)	0.58
Diabetes with complications	213 (57.7%)	208 (58.6%)	5 (35.7%)	0.089	187 (57.4%)	179 (58.5%)	8 (40.0%)	0.11
Renal disease	355 (96.2%)	342 (96.3%)	13 (92.9%)	0.5	315 (96.6%)	297 (97.1%)	18 (90.0%)	0.09
Peptic ulcer disease	35 (9.5%)	33 (9.3%)	2 (14.3%)	0.53	30 (9.2%)	27 (8.8%)	3 (15.0%)	0.35
Mild liver disease	118 (32.0%)	115 (32.4%)	3 (21.4%)	0.39	109 (33.4%)	104 (34.0%)	5 (25.0%)	0.41
Moderate to severe liver disease	62 (16.8%)	57 (16.1%)	5 (35.7%)	0.054	50 (15.3%)	43 (14.1%)	7 (35.0%)	0.012
Dementia	3 (0.8%)	3 (0.8%)	0 (0.0%)	0.73	3 (0.9%)	3 (1.0%)	0 (0.0%)	0.66
Hemiplegia	18 (4.9%)	18 (5.1%)	0 (0.0%)	0.39	16 (4.9%)	15 (4.9%)	1 (5.0%)	0.98
Rheumatoid disease	27 (7.3%)	27 (7.6%)	0 (0.0%)	0.28	24 (7.4%)	23 (7.5%)	1 (5.0%)	0.68
Cancer	61 (16.5%)	59 (16.6%)	2 (14.3%)	0.82	52 (16.0%)	50 (16.3%)	2 (10.0%)	0.45
Metastatic cancer	42 (11.4%)	41 (11.5%)	1 (7.1%)	0.61	34 (10.4%)	32 (10.5%)	2 (10.0%)	0.95
HIV/AIDS	4 (1.1%)	4 (1.1%)	0 (0.0%)	0.69	4 (1.2%)	4 (1.3%)	0 (0.0%)	0.61
Transplant characteristics								
Transplanted organ				0.36				0.036
Kidney	272 (73.7%)	263 (74.1%)	9 (64.3%)		240 (73.6%)	230 (75.2%)	10 (50.0%)	
Liver	52 (14.1%)	48 (13.5%)	4 (28.6%)		43 (13.2%)	37 (12.1%)	6 (30.0%)	
Heart	12 (3.3%)	11 (3.1%)	1 (7.1%)		10 (3.1%)	8 (2.6%)	2 (10.0%)	
Lung	24 (6.5%)	24 (6.8%)	0 (0.0%)		24 (7.4%)	22 (7.2%)	2 (10.0%)	
Heart–lung	0 (0.0%)	0 (0.0%)	0 (0.0%)		0 (0.0%)	0 (0.0%)	0 (0.0%)	
Pancreas	0 (0.0%)	0 (0.0%)	0 (0.0%)		0 (0.0%)	0 (0.0%)	0 (0.0%)	
Kidney–pancreas	9 (2.4%)	9 (2.5%)	0 (0.0%)		9 (2.8%)	9 (2.9%)	0 (0.0%)	
Other (includes islet)	0 (0.0%)	0 (0.0%)	0 (0.0%)		0 (0.0%)	0 (0.0%)	0 (0.0%)	
Donor type				0.55				0.21
Deceased donor (brain death)	133 (36.0%)	127 (35.8%)	6 (42.9%)		123 (37.7%)	112 (36.6%)	11 (55.0%)	
Deceased donor (cardiac death)	31 (8.4%)	29 (8.2%)	2 (14.3%)		28 (8.6%)	26 (8.5%)	2 (10.0%)	
Living donor	205 (55.6%)	199 (56.1%)	6 (42.9%)		175 (53.7%)	168 (54.9%)	7 (35.0%)	
COVID-19 characteristics								
Date of the first recorded COVID-19 diagnosis (by local peak)				0.8				0.052
1 February 2020–15 September 2020	6 (1.6%)	5 (1.4%)	1 (7.1%)		6 (1.8%)	4 (1.3%)	2 (10.0%)	
16 September 2020–20 June 2021	59 (16.0%)	57 (16.1%)	2 (14.3%)		59 (18.1%)	53 (17.3%)	6 (30.0%)	
21 June 2021–20 November 2021	55 (14.9%)	53 (14.9%)	2 (14.3%)		55 (16.9%)	52 (17.0%)	3 (15.0%)	
21 November 2021–1 April 2022	53 (14.4%)	50 (14.1%)	3 (21.4%)		52 (16.0%)	48 (15.7%)	4 (20.0%)	
2 April 2022–15 October 2022	61 (16.5%)	60 (16.9%)	1 (7.1%)		61 (18.7%)	60 (19.6%)	1 (5.0%)	
16 October 2022–1 June 2023	60 (16.3%)	57 (16.1%)	3 (21.4%)		60 (18.4%)	57 (18.6%)	3 (15.0%)	
2 June 2023–15 November 2023	33 (8.9%)	32 (9.0%)	1 (7.1%)		33 (10.1%)	32 (10.5%)	1 (5.0%)	
16 November 2023–1 May 2024	38 (10.3%)	37 (10.4%)	1 (7.1%)		0 (0.0%)	0 (0.0%)	0 (0.0%)	
2 May 2024–15 December 2024	4 (1.1%)	4 (1.1%)	0 (0.0%)		0 (0.0%)	0 (0.0%)	0 (0.0%)	
Status at the first recorded COVID-19 diagnostic encounter								
Hospitalized within 30 days	149 (40.4%)	143 (40.3%)	6 (42.9%)	0.85	135 (41.4%)	125 (40.8%)	10 (50.0%)	0.42
Intensive care admission within 30 days	79 (21.4%)	75 (21.1%)	4 (28.6%)	0.51	69 (21.2%)	62 (20.3%)	7 (35.0%)	0.12

Differences across exposure groups compared using Fisher's exact tests (categorical variables) or Kruskal–Wallis tests (continuous variables).

Includes patients whose first recorded COVID-19 laboratory test positive was within 30 days +/− of transplant. Exposures: demographic and clinical variables abstracted from EMR at the first COVID-19 diagnostic encounter. Outcome: composite all-cause mortality or graft failure within 90 days or 1 year of transplant date. Patients who received transplants within 90 days (Outcome A) or 1 year (Outcome B) of the study end date and those lost to follow-up were excluded.

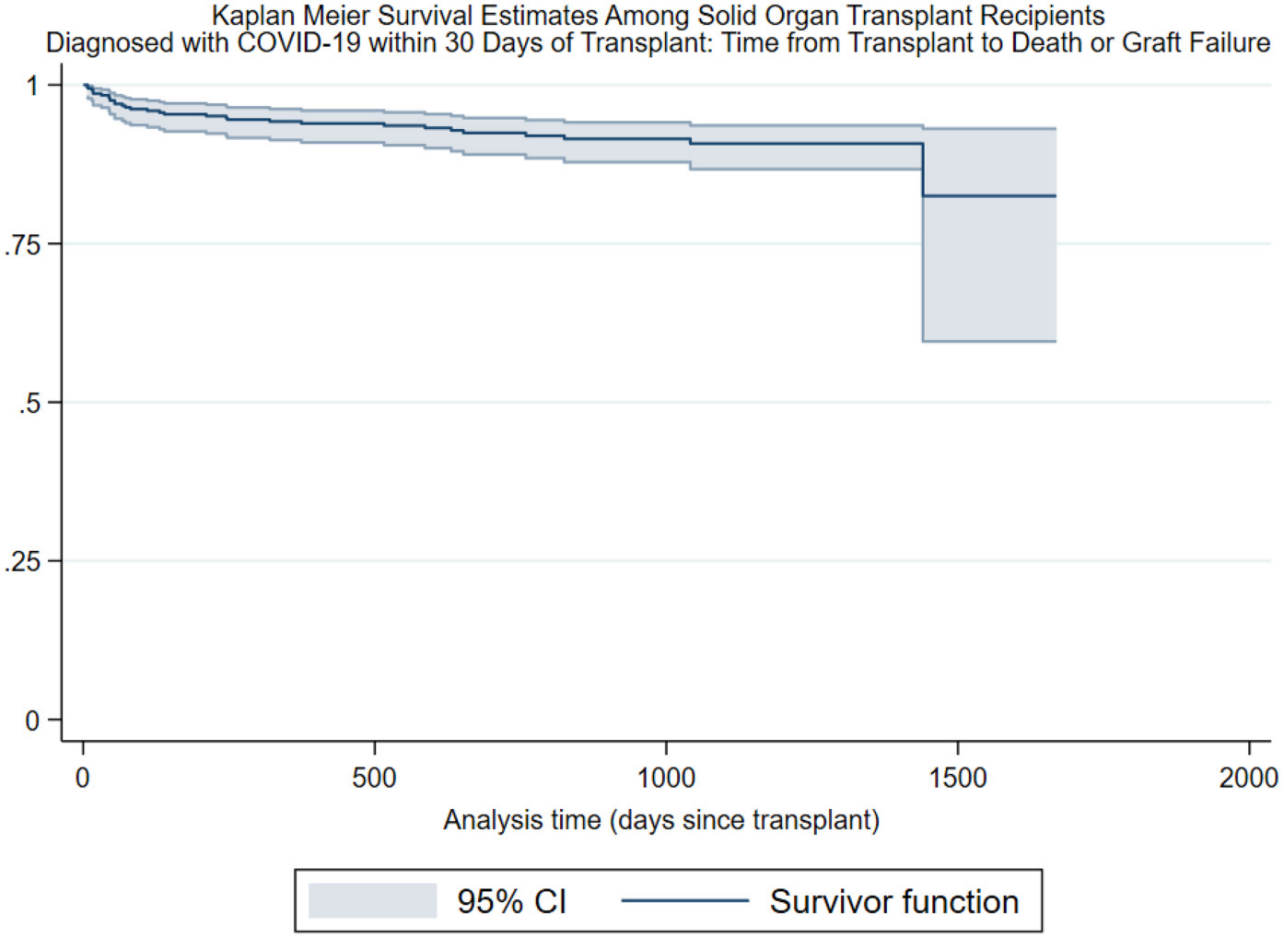

Legend: Kaplan–Meier analyses, the *y*-axis represents survivorship, the *x*-axis represents analysis, time zero was the date of transplant, and composite events were recorded as the date of death or graft failure; patients were right-censored at study end or date of loss to follow-up. CI, confidence interval.

Survival function at 90 days: 0.96, CI: 0.94–0.98.

Survival function at 365 days: 0.94, CI: 0.91–0.96.

### 3.5 Post-SOT COVID-19

Table 6 compares the composite outcome of death or graft failure post COVID-19 diagnosis among patients whose first COVID-19 episode was recorded >30 days after their transplant date (90 days post COVID-19, *N* = 2,029, 122 events; 6–18 months post COVID-19, *N* = 1,065, 73 events). Increasing age, Medicare/Medicaid payor, myocardial infarction history, congestive heart failure, peripheral vascular disease, severe diabetes, renal disease, COVID-19 diagnosed in the first wave (1 February 2020 to 15 September 2020), and hospitalization within 30 days of COVID-19 diagnosis were associated with composite outcomes in the 90 days post COVID-19 diagnosis. Among patients who survived at least 6 months post initial COVID-19 diagnosis, increasing age, chronic obstructive pulmonary disease, myocardial infarction history, congestive heart failure, peripheral vascular disease, cerebrovascular disease, and severe diabetes were associated with death or graft failure in the 6–18 months post COVID-19. Lung transplant, COVID-19 diagnosed in the period 16 October 2022 to 1 June 2023, and hospitalization within 30 days of COVID-19 diagnosis were additionally associated with composite outcomes in the 6–18 months post COVID-19 diagnosis. Long COVID was not associated with death/graft failure, 6–18 months post COVID-19.

**Table 6:** Ninety-day and 1-year post COVID-19 diagnosis death/graft failure among solid organ transplant recipients diagnosed with COVID-19 >30 days posttransplant.

Characteristics	(A) Death/graft failure: 90 days post-first COVID				(B) Death/graft failure: 1 year post-first COVID			
	Total *N* = 2,029	No events, *n* = 1,907	Events, *n* = 122	*p*-value	Total *n* = 1,906	No event, *n* = 1,697	Events, *n* = 209	*p*-value
Demographics at the first reported COVID-19 episode	*n* (%)	*n* (%)	*n* (%)		*n* (%)	*n* (%)	*n* (%)	
Age at encounter (years), median (IQR)	60 (50–68)	60 (49–67)	65 (53–70)	0.002	60 (49–67)	59 (49–67)	65 (55–71)	<0.001
Gender				0.26				0.1
Female	848 (41.8%)	791 (41.5%)	57 (46.7%)		789 (41.4%)	691 (40.7%)	98 (46.9%)	
Male	1,181 (58.2%)	1,116 (58.5%)	65 (53.3%)		1,117 (58.6%)	1,006 (59.3%)	111 (53.1%)	
Race/ethnicity				0.15				0.02
Non-Hispanic White	1,007 (49.6%)	955 (50.1%)	52 (42.6%)		950 (49.8%)	847 (49.9%)	103 (49.3%)	
Non-Hispanic Black	415 (20.5%)	382 (20.0%)	33 (27.0%)		391 (20.5%)	333 (19.6%)	58 (27.8%)	
Non-Hispanic Asian	116 (5.7%)	113 (5.9%)	3 (2.5%)		108 (5.7%)	104 (6.1%)	4 (1.9%)	
Non-Hispanic Hawaiian/Pacific	5 (0.2%)	5 (0.3%)	0 (0.0%)		5 (0.3%)	5 (0.3%)	0 (0.0%)	
Non-Hispanic Native American	7 (0.3%)	6 (0.3%)	1 (0.8%)		7 (0.4%)	6 (0.4%)	1 (0.5%)	
Non-Hispanic other race	0 (0.0%)	0 (0.0%)	0 (0.0%)		0 (0.0%)	0 (0.0%)	0 (0.0%)	
Hispanic or Latino	470 (23.2%)	437 (22.9%)	33 (27.0%)		438 (23.0%)	396 (23.3%)	42 (20.1%)	
Unknown	9 (0.4%)	9 (0.5%)	0 (0.0%)		7 (0.4%)	6 (0.4%)	1 (0.5%)	
Area deprivation index (state rank)				0.27				0.13
1–2 (least deprivation)	549 (27.1%)	525 (27.5%)	24 (19.7%)		521 (27.3%)	473 (27.9%)	48 (23.0%)	
3–4	499 (24.6%)	471 (24.7%)	28 (23.0%)		468 (24.6%)	423 (24.9%)	45 (21.5%)	
5–6	394 (19.4%)	368 (19.3%)	26 (21.3%)		368 (19.3%)	327 (19.3%)	41 (19.6%)	
7–8	341 (16.8%)	314 (16.5%)	27 (22.1%)		319 (16.7%)	271 (16.0%)	48 (23.0%)	
9–10 (most deprivation)	216 (10.6%)	200 (10.5%)	16 (13.1%)		202 (10.6%)	177 (10.4%)	25 (12.0%)	
Missing	30 (1.5%)	29 (1.5%)	1 (0.8%)		28 (1.5%)	26 (1.5%)	2 (1.0%)	
Financial class at the first COVID-19 diagnosis				<0.001				<0.001
Private insurance	730 (36.0%)	706 (37.0%)	24 (19.7%)		699 (36.7%)	654 (38.5%)	45 (21.5%)	
Medicare/Medicaid	1,231 (60.7%)	1,136 (59.6%)	95 (77.9%)		1,150 (60.3%)	992 (58.5%)	158 (75.6%)	
Self-pay	23 (1.1%)	22 (1.2%)	1 (0.8%)		21 (1.1%)	18 (1.1%)	3 (1.4%)	
Other	16 (0.8%)	14 (0.7%)	2 (1.6%)		16 (0.8%)	14 (0.8%)	2 (1.0%)	
Missing	29 (1.4%)	29 (1.5%)	0 (0.0%)		20 (1.0%)	19 (1.1%)	1 (0.5%)	
Clinical history (at the first COVID-19 diagnosis)								
Body mass index				0.5				0.63
BMI ≤ 18.5	26 (1.3%)	21 (1.1%)	5 (4.1%)		24 (1.3%)	17 (1.0%)	7 (3.3%)	
BMI = 18.5–25	260 (12.8%)	228 (12.0%)	32 (26.2%)		234 (12.3%)	181 (10.7%)	53 (25.4%)	
BMI = 25–30	302 (14.9%)	268 (14.1%)	34 (27.9%)		258 (13.5%)	208 (12.3%)	50 (23.9%)	
BMI = 30–35	188 (9.3%)	167 (8.8%)	21 (17.2%)		165 (8.7%)	137 (8.1%)	28 (13.4%)	
BMI = 35–40	64 (3.2%)	52 (2.7%)	12 (9.8%)		60 (3.1%)	47 (2.8%)	13 (6.2%)	
BMI > 40	34 (1.7%)	30 (1.6%)	4 (3.3%)		30 (1.6%)	24 (1.4%)	6 (2.9%)	
Missing	1,155 (56.9%)	1,141 (59.8%)	14 (11.5%)		1,135 (59.5%)	1,083 (63.8%)	52 (24.9%)	
Charlson comorbidity index components								
Asthma	294 (14.5%)	279 (14.6%)	15 (12.3%)	0.48	275 (14.4%)	237 (14.0%)	38 (18.2%)	0.1
COPD	954 (47.0%)	898 (47.1%)	56 (45.9%)	0.8	903 (47.4%)	784 (46.2%)	119 (56.9%)	0.003
Tuberculosis	91 (4.5%)	85 (4.5%)	6 (4.9%)	0.81	88 (4.6%)	75 (4.4%)	13 (6.2%)	0.24
Myocardial infarction	755 (37.2%)	688 (36.1%)	67 (54.9%)	<0.001	717 (37.6%)	599 (35.3%)	118 (56.5%)	<0.001
Congestive heart failure	1,095 (54.0%)	1,016 (53.3%)	79 (64.8%)	0.014	1,024 (53.7%)	875 (51.6%)	149 (71.3%)	<0.001
Peripheral vascular disease	1,401 (69.0%)	1,307 (68.5%)	94 (77.0%)	0.049	1,315 (69.0%)	1,151 (67.8%)	164 (78.5%)	0.002
Cerebrovascular disease	1,025 (50.5%)	971 (50.9%)	54 (44.3%)	0.15	951 (49.9%)	838 (49.4%)	113 (54.1%)	0.2
Diabetes without complications	202 (10.0%)	192 (10.1%)	10 (8.2%)	0.5	197 (10.3%)	185 (10.9%)	12 (5.7%)	0.021
Diabetes with complications	1,352 (66.6%)	1,260 (66.1%)	92 (75.4%)	0.034	1,262 (66.2%)	1,095 (64.5%)	167 (79.9%)	<0.001
Renal disease	1,931 (95.2%)	1,810 (94.9%)	121 (99.2%)	0.033	1,811 (95.0%)	1,606 (94.6%)	205 (98.1%)	0.031
Peptic ulcer disease	251 (12.4%)	235 (12.3%)	16 (13.1%)	0.8	235 (12.3%)	204 (12.0%)	31 (14.8%)	0.24
Mild liver disease	665 (32.8%)	633 (33.2%)	32 (26.2%)	0.11	636 (33.4%)	570 (33.6%)	66 (31.6%)	0.56
Moderate to severe liver disease	527 (26.0%)	504 (26.4%)	23 (18.9%)	0.064	477 (25.0%)	437 (25.8%)	40 (19.1%)	0.037
Dementia	77 (3.8%)	69 (3.6%)	8 (6.6%)	0.1	73 (3.8%)	56 (3.3%)	17 (8.1%)	<0.001
Hemiplegia	134 (6.6%)	121 (6.3%)	13 (10.7%)	0.063	121 (6.3%)	99 (5.8%)	22 (10.5%)	0.009
Rheumatoid disease	252 (12.4%)	239 (12.5%)	13 (10.7%)	0.54	234 (12.3%)	207 (12.2%)	27 (12.9%)	0.76
Cancer	588 (29.0%)	562 (29.5%)	26 (21.3%)	0.054	549 (28.8%)	500 (29.5%)	49 (23.4%)	0.07
Metastatic cancer	290 (14.3%)	271 (14.2%)	19 (15.6%)	0.68	263 (13.8%)	231 (13.6%)	32 (15.3%)	0.5
HIV/AIDS	29 (1.4%)	29 (1.5%)	0 (0.0%)	0.17	26 (1.4%)	25 (1.5%)	1 (0.5%)	0.24
Transplant characteristics								
Transplanted organ				0.055				<0.001
Kidney	758 (37.4%)	697 (36.5%)	61 (50.0%)		716 (37.6%)	633 (37.3%)	83 (39.7%)	
Liver	517 (25.5%)	496 (26.0%)	21 (17.2%)		470 (24.7%)	438 (25.8%)	32 (15.3%)	
Heart	253 (12.5%)	238 (12.5%)	15 (12.3%)		234 (12.3%)	211 (12.4%)	23 (11.0%)	
Lung	371 (18.3%)	348 (18.2%)	23 (18.9%)		359 (18.8%)	298 (17.6%)	61 (29.2%)	
Heart–lung	20 (1.0%)	20 (1.0%)	0 (0.0%)		20 (1.0%)	17 (1.0%)	3 (1.4%)	
Pancreas	6 (0.3%)	6 (0.3%)	0 (0.0%)		6 (0.3%)	6 (0.4%)	0 (0.0%)	
Kidney–pancreas	103 (5.1%)	101 (5.3%)	2 (1.6%)		100 (5.2%)	93 (5.5%)	7 (3.3%)	
Other (includes islet)	1 (0.0%)	1 (0.1%)	0 (0.0%)		1 (0.1%)	1 (0.1%)	0 (0.0%)	
Donor type				0.11				0.066
Deceased donor (brain death)	1,557 (76.7%)	1,464 (76.8%)	93 (76.2%)		1,458 (76.5%)	1,294 (76.3%)	164 (78.5%)	
Deceased donor (cardiac death)	91 (4.5%)	81 (4.2%)	10 (8.2%)		82 (4.3%)	68 (4.0%)	14 (6.7%)	
Living donor	353 (17.4%)	338 (17.7%)	15 (12.3%)		339 (17.8%)	313 (18.4%)	26 (12.4%)	
Missing	28 (1.4%)	24 (1.3%)	4 (3.3%)		27 (1.4%)	22 (1.3%)	5 (2.4%)	
Year of most recent transplant				0.16				0.19
Before 2018	1,020 (50.3%)	947 (49.7%)	73 (59.8%)		985 (51.7%)	861 (50.7%)	124 (59.3%)	
2018	199 (9.8%)	193 (10.1%)	6 (4.9%)		193 (10.1%)	175 (10.3%)	18 (8.6%)	
2019	254 (12.5%)	236 (12.4%)	18 (14.8%)		243 (12.7%)	220 (13.0%)	23 (11.0%)	
2020	299 (14.7%)	284 (14.9%)	15 (12.3%)		293 (15.4%)	272 (16.0%)	21 (10.0%)	
2021	139 (6.9%)	131 (6.9%)	8 (6.6%)		130 (6.8%)	113 (6.7%)	17 (8.1%)	
2022	63 (3.1%)	62 (3.3%)	1 (0.8%)		53 (2.8%)	48 (2.8%)	5 (2.4%)	
2023	49 (2.4%)	48 (2.5%)	1 (0.8%)		9 (0.5%)	8 (0.5%)	1 (0.5%)	
2024	6 (0.3%)	6 (0.3%)	0 (0.0%)		0 (0.0%)	0 (0.0%)	0 (0.0%)	
COVID-19 characteristics								
Date of the first recorded COVID-19 diagnosis (by local peak)				<0.001				<0.001
1 February 2020–15 September 2020	132 (6.5%)	111 (5.8%)	21 (17.2%)		132 (6.9%)	106 (6.2%)	26 (12.4%)	
16 September 2020–20 June 2021	751 (37.0%)	720 (37.8%)	31 (25.4%)		750 (39.3%)	695 (41.0%)	55 (26.3%)	
21 June 2021–20 November 2021	344 (17.0%)	324 (17.0%)	20 (16.4%)		344 (18.0%)	310 (18.3%)	34 (16.3%)	
21 November 2021–1 April 2022	343 (16.9%)	320 (16.8%)	23 (18.9%)		341 (17.9%)	295 (17.4%)	46 (22.0%)	
2 April 2022–15 October 2022	169 (8.3%)	160 (8.4%)	9 (7.4%)		168 (8.8%)	149 (8.8%)	19 (9.1%)	
16 October 2022–1 June 2023	106 (5.2%)	99 (5.2%)	7 (5.7%)		105 (5.5%)	89 (5.2%)	16 (7.7%)	
2 June 2023–15 November 2023	56 (2.8%)	51 (2.7%)	5 (4.1%)		56 (2.9%)	47 (2.8%)	9 (4.3%)	
16 November 2023–1 May 2024	76 (3.7%)	71 (3.7%)	5 (4.1%)		10 (0.5%)	6 (0.4%)	4 (1.9%)	
2 May 2024–15 December 2024	52 (2.6%)	51 (2.7%)	1 (0.8%)		0 (0.0%)	0 (0.0%)	0 (0.0%)	
Status at the first recorded COVID-19 diagnostic encounter								
Hospitalized within 30 days	704 (34.7%)	604 (31.7%)	100 (82.0%)	<0.001	631 (33.1%)	487 (28.7%)	144 (68.9%)	<0.001
Intensive care admission within 30 days	146 (7.2%)	76 (4.0%)	70 (57.4%)	<0.001	138 (7.2%)	59 (3.5%)	79 (37.8%)	<0.001
Long COVID diagnosis	Not included				92 (4.8%)	90 (5.3%)	2 (1.0%)	0.006

Differences across exposure groups compared using Fisher's exact tests (categorical variables) or Kruskal–Wallis tests (continuous variables).

Outcome A: includes patients whose first recorded COVID-19 laboratory test positive was >30 days following transplant. Exposures: demographic and clinical variables abstracted from EMR at the first COVID-19 diagnostic encounter. Outcome: composite all-cause mortality or graft failure within 90 days of the first recorded COVID-19 diagnosis date. Patients with a first recorded COVID-19 diagnosis date within 90 days of the study end date and those lost to follow-up were excluded.

Outcome B: includes patients whose first recorded COVID-19 laboratory test positive was >30 days following transplant and who survived at least 180 days following their first COVID-19 laboratory test positive. Exposures: demographic and clinical variables abstracted from EMR at the first COVID-19 diagnostic encounter. Long COVID diagnosis in the 180 days following the first COVID-19 laboratory test positive was the primary exposure. Outcome: composite all-cause mortality or graft failure between 6 months and 18 months of the first recorded COVID-19 diagnosis date. Patients with a first recorded COVID-19 diagnosis date within 18 months of the study end date and those lost to follow-up were excluded. Patients with a first recorded COVID-19 diagnosis date before 1 April 2021 were excluded, as they could not have received a long COVID diagnosis prior to the observation period.

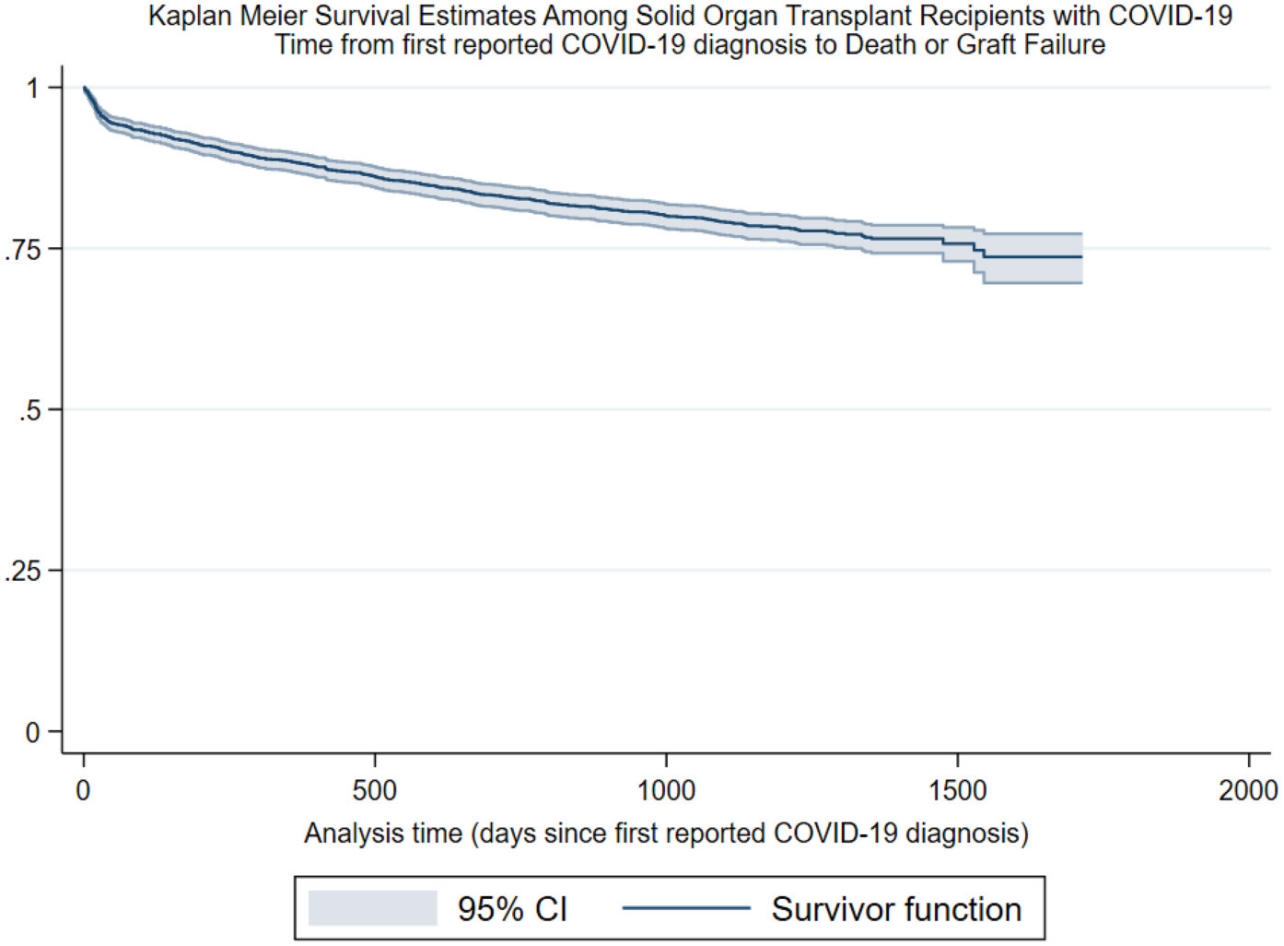

Legend: Kaplan–Meier analyses, the *y*-axis represents survivorship, the *x*-axis represents analysis, time zero was the date of the first recorded COVID-19 laboratory test positive, and composite events were recorded as the date of death or graft failure; patients were right-censored at study end or date of loss to follow-up. CI, confidence interval.

Survival function at 90 days: 0.93, CI: 0.92–0.95.

### 3.6 Supplementary analyses

In supplementary analyses, patients who received SOT between 2020 and 2024 but who did not have a recorded COVID-19 diagnosis were examined as a baseline population (Supplementary Table S2). Deceased donor type vs. brain death donor type and lung transplant vs. other organ transplant were associated with the 1-year death/graft failure composite outcome (*N* = 1,082, 94 events). Characteristics of SOT recipients diagnosed with long COVID are presented in Supplementary Table S3.

## 4 Discussion

Our findings add to the growing body of literature demonstrating the multifaceted risk profile of both acute COVID-19 and long COVID among solid organ transplant recipients. We identified distinct subpopulations of patients who had COVID-19 before their transplants, those whose COVID-19 occurred around the time of their transplant, and those who contracted COVID-19 after receiving SOT; these sub-cohorts exhibit discrete clinical trajectories and provide specific insights for future epidemiologic studies. Among patients diagnosed with COVID-19 before their transplants, overall mortality was ∼8%, but mortality among those who were eventually diagnosed with long COVID was 20%, suggesting that poor recovery from COVID-19 may impact subsequent transplant outcomes (Figure 1). COVID-19 in the immediate transplant period, which was likely captured at a pretransplant screening or during recovery, did not appear to increase baseline mortality. However, in the group of patients who were diagnosed with COVID-19 posttransplant, the mortality rate due to acute COVID-19 was high (5.4% expired within 90 days of COVID-19 diagnosis, 110/2,029) and associated with an increased comorbidity burden, consistent with extant literature (12–14). The role of long COVID in extended morbidity and mortality in this population is more difficult to interpret, as acute COVID-19 and transplant complications act as competing risks. Notably, long COVID diagnoses were most common among lung transplant recipients: 40% (49/122) of patients diagnosed with long COVID after transplant had received lung transplants. Long COVID may act as a mediator or moderator of mortality risk in this population with increased vulnerability to infectious disease and high comorbidity burden (35).

This investigation was limited to descriptive analyses of clinical and demographic risk factors, so causal relationships cannot be demonstrated. However, the findings are relevant in shaping future investigations into long COVID as both a risk factor and an outcome among solid organ transplant recipients. As with all electronic health record-based studies, some clinical parameters were not available in our dataset, including vaccination history, COVID-19 episodes undetected by the health system, and detailed pharmacology records. Our primary exposure was long COVID diagnosis, determined by the diagnostic code U09.9, which was introduced by the World Health Organization (WHO) in October 2021 to specifically identify long COVID cases; some patients who exhibited symptoms associated with long COVID before the fall of 2021 may therefore have been misclassified as long COVID-negative. The diagnosis of long COVID may be influenced by multiple factors, including access to healthcare, clinical history, and specific symptom presentation. We were not able to independently ascertain long COVID status outside of the presence or absence of the diagnostic code, and indeed, the most appropriate strategy to determine long COVID status would include prospective, longitudinal, active follow-up via survey or study-specific clinical encounters. However, the population of SOT recipients is universally subject to extended clinical follow-up as part of their posttransplant care, which likely reduced the proportion of unobserved long COVID symptomology in this cohort. An additional limitation was our inability to incorporate multiple COVID-19 episodes per patient, which could have allowed us to construct composite risk profiles integrating SARS-CoV-2 variants of concern and recurrent vs. persistent viral infections. Missingness in BMI was high; however, given the extensive clinical follow-up in SOT recipients, this missingness is most likely due to challenges in data extraction, rather than systematic bias, though poor coverage may limit interpretation of this result. Our study's strengths are a robust sample size, a diverse, heterogeneous population, and significant longitudinal follow-up of SOT patients. This investigation was a single-center, registry-based chart review, so patients could have unobserved COVID-19 or long COVID diagnoses from other health systems; however, the SOT recipient population is closely monitored by the transplant team, including SARS-CoV-2 testing for surveillance purposes, regardless of symptoms. Importantly, the outcomes of death and graft failure were abstracted directly from the national UNOS registry, mitigating information loss between health systems.

The observed differences between patients diagnosed with COVID-19 and long COVID before and after transplant warrant additional studies as the proportion of people with some SARS-CoV-2 infection history approaches 90% (36). Future investigations may prioritize longitudinal follow-up of long COVID patients diagnosed before or after transplant to determine specific etiologies of long-term morbidity and mortality.

## Data Availability

Data cannot be shared publicly because of patient confidentiality and intellectual property concerns as imposed by the Houston Methodist Research Institute. Access to de-identified data can be made to the Director of the Center for Health Data Science and Analytics at Houston Methodist Research Institute (chdsa@houstonmethodist.org). Each request will be evaluated on a case-by-case basis in line with institutional policies.
