# Supplementary material for: Long COVID risk factors and outcomes among solid organ transplant recipients: a retrospective cohort study
Source: Front Surg. 2025 Aug 25;12:1602167. doi: 10.3389/fsurg.2025.1602167 (PMC12415067; doi:10.3389/fsurg.2025.1602167)
Supplement: Supplementary file 2 [file Table2.docx]

Supplemental analyses

| Table S.1.1: 90-day COVID survivor solid organ transplant recipients by long COVID status, October 2021-November 2024 | | | | |
| --- | --- | --- | --- | --- |
|  |  |  |  |  |
|  | Total | No Long COVID | Long COVID |  |
| **Characteristics** | N=837 | N=789 | N=48 | p-value |
| **Demographics at first reported COVID-19 episode** | *n (%)* | *n (%)* | *n (%)* |  |
| Age at encounter (years), median (IQR) | 61 (50-68) | 60 (50-68) | 64.5 (54-69) | 0.27 |
| Gender |  |  |  | 0.88 |
| Female | 356 (42.5%) | 335 (42.5%) | 21 (43.8%) |  |
| Male | 481 (57.5%) | 454 (57.5%) | 27 (56.3%) |  |
| Race/Ethnicity |  |  |  | 0.77 |
| NH White | 405 (48.4%) | 381 (48.3%) | 24 (50.0%) |  |
| NH Black | 198 (23.7%) | 187 (23.7%) | 11 (22.9%) |  |
| NH Asian | 39 (4.7%) | 38 (4.8%) | 1 (2.1%) |  |
| NH Hawaiian/Pacific | 3 (0.4%) | 3 (0.4%) | 0 (0.0%) |  |
| NH Native American | 2 (0.2%) | 2 (0.3%) | 0 (0.0%) |  |
| NH Other Race | 0 (0.0%) | 0 (0.0%) | 0 (0.0%) |  |
| Hispanic or Latino | 183 (21.9%) | 172 (21.8%) | 11 (22.9%) |  |
| Unknown | 7 (0.8%) | 6 (0.8%) | 1 (2.1%) |  |
| Area Deprivation Index (state rank) |  |  |  | 0.76 |
| 1 to 2 (Least deprivation) | 227 (27.1%) | 213 (27.0%) | 14 (29.2%) |  |
| 3 to 4 | 207 (24.7%) | 197 (25.0%) | 10 (20.8%) |  |
| 5 to 6 | 159 (19.0%) | 146 (18.5%) | 13 (27.1%) |  |
| 7 to 8 | 153 (18.3%) | 146 (18.5%) | 7 (14.6%) |  |
| 9 to 10 (Most deprivation) | 80 (9.6%) | 76 (9.6%) | 4 (8.3%) |  |
| Missing | 11 (1.3%) | 11 (1.4%) | 0 (0.0%) |  |
| Financial Class at first COVID-19 diagnosis |  |  |  | 0.67 |
| Private insurance | 279 (33.3%) | 266 (33.7%) | 13 (27.1%) |  |
| Medicare/Medicaid | 523 (62.5%) | 488 (61.9%) | 35 (72.9%) |  |
| Self-Pay | 9 (1.1%) | 9 (1.1%) | 0 (0.0%) |  |
| Other | 8 (1.0%) | 8 (1.0%) | 0 (0.0%) |  |
| Missing | 18 (2.2%) | 18 (2.3%) | 0 (0.0%) |  |
| **Clinical history (at first COVID-19 diagnosis)** | | | | |
| Body mass index |  |  |  | 0.18 |
| BMI <=18.5 | 15 (1.8%) | 14 (1.8%) | 1 (2.1%) |  |
| BMI 18.5-25 | 143 (17.1%) | 138 (17.5%) | 5 (10.4%) |  |
| BMI 25-30 | 170 (20.3%) | 155 (19.6%) | 15 (31.3%) |  |
| BMI 30-35 | 116 (13.9%) | 112 (14.2%) | 4 (8.3%) |  |
| BMI 35-40 | 31 (3.7%) | 28 (3.5%) | 3 (6.3%) |  |
| BMI >40 | 16 (1.9%) | 16 (2.0%) | 0 (0.0%) |  |
| Missing | 346 (41.3%) | 326 (41.3%) | 20 (41.7%) |  |
| Charlson Comorbidity Index Score |  |  |  | 0.73 |
| Score 1-2 | 16 (1.9%) | 16 (2.0%) | 0 (0.0%) |  |
| Score 3-4 | 50 (6.0%) | 48 (6.1%) | 2 (4.2%) |  |
| Score >4 | 771 (92.1%) | 725 (91.9%) | 46 (95.8%) |  |
| Charlson Comorbidity Index Components |  |  |  |  |
| Asthma | 119 (14.2%) | 111 (14.1%) | 8 (16.7%) | 0.62 |
| COPD | 393 (47.0%) | 360 (45.6%) | 33 (68.8%) | 0.002 |
| Tuberculosis | 37 (4.4%) | 34 (4.3%) | 3 (6.3%) | 0.53 |
| Myocardial Infarction | 318 (38.0%) | 294 (37.3%) | 24 (50.0%) | 0.078 |
| Congestive heart failure | 484 (57.8%) | 449 (56.9%) | 35 (72.9%) | 0.029 |
| Peripheral vascular disease | 613 (73.2%) | 573 (72.6%) | 40 (83.3%) | 0.1 |
| Cerebrovascular disease | 478 (57.1%) | 443 (56.1%) | 35 (72.9%) | 0.023 |
| Diabetes without complications | 613 (73.2%) | 573 (72.6%) | 40 (83.3%) | 0.1 |
| Diabetes with complications | 574 (68.6%) | 537 (68.1%) | 37 (77.1%) | 0.19 |
| Renal disease | 791 (94.5%) | 745 (94.4%) | 46 (95.8%) | 0.68 |
| Peptic ulcer disease | 111 (13.3%) | 104 (13.2%) | 7 (14.6%) | 0.78 |
| Mild liver disease | 502 (60.0%) | 473 (59.9%) | 29 (60.4%) | 0.95 |
| Moderate to severe liver disease | 254 (30.3%) | 244 (30.9%) | 10 (20.8%) | 0.14 |
| Dementia | 30 (3.6%) | 30 (3.8%) | 0 (0.0%) | 0.17 |
| Hemiplegia | 62 (7.4%) | 57 (7.2%) | 5 (10.4%) | 0.41 |
| Rheumatoid disease | 107 (12.8%) | 98 (12.4%) | 9 (18.8%) | 0.2 |
| Cancer | 222 (26.5%) | 208 (26.4%) | 14 (29.2%) | 0.67 |
| Metastatic cancer | 125 (14.9%) | 118 (15.0%) | 7 (14.6%) | 0.94 |
| HIV/AIDS | 13 (1.6%) | 13 (1.6%) | 0 (0.0%) | 0.37 |
| **Transplant characteristics** | | | | |
| Transplanted organ |  |  |  | 0.052 |
| Kidney | 285 (34.1%) | 272 (34.5%) | 13 (27.1%) |  |
| Liver | 224 (26.8%) | 216 (27.4%) | 8 (16.7%) |  |
| Heart | 128 (15.3%) | 117 (14.8%) | 11 (22.9%) |  |
| Lung | 146 (17.4%) | 131 (16.6%) | 15 (31.3%) |  |
| Heart-Lung | 7 (0.8%) | 6 (0.8%) | 1 (2.1%) |  |
| Pancreas | 3 (0.4%) | 3 (0.4%) | 0 (0.0%) |  |
| Kidney-Pancreas | 43 (5.1%) | 43 (5.4%) | 0 (0.0%) |  |
| Other (Includes Islet) | 1 (0.1%) | 1 (0.1%) | 0 (0.0%) |  |
| Donor type |  |  |  | 0.54 |
| Deceased donor (brain death) | 648 (77.4%) | 608 (77.1%) | 40 (83.3%) |  |
| Deceased donor (cardiac death) | 53 (6.3%) | 51 (6.5%) | 2 (4.2%) |  |
| Living donor | 123 (14.7%) | 118 (15.0%) | 5 (10.4%) |  |
| Unknown | 13 (1.6%) | 12 (1.5%) | 1 (2.1%) |  |
| Year of most recent transplant |  |  |  | 0.58 |
| before 2018 | 284 (33.9%) | 265 (33.6%) | 19 (39.6%) |  |
| 2018 | 78 (9.3%) | 73 (9.3%) | 5 (10.4%) |  |
| 2019 | 91 (10.9%) | 90 (11.4%) | 1 (2.1%) |  |
| 2020 | 113 (13.5%) | 107 (13.6%) | 6 (12.5%) |  |
| 2021 | 111 (13.3%) | 103 (13.1%) | 8 (16.7%) |  |
| 2022 | 90 (10.8%) | 84 (10.6%) | 6 (12.5%) |  |
| 2023 | 56 (6.7%) | 53 (6.7%) | 3 (6.3%) |  |
| 2024 | 14 (1.7%) | 14 (1.8%) | 0 (0.0%) |  |
| Transplant outcome (first recorded event) |  |  |  | 0.14 |
| Death | 91 (10.9%) | 88 (11.2%) | 3 (6.3%) |  |
| Graft failure | 31 (3.7%) | 27 (3.4%) | 4 (8.3%) |  |
| Loss to follow-up | 18 (2.2%) | 17 (2.2%) | 1 (2.1%) |  |
| Remained under observation | 697 (83.3%) | 657 (83.3%) | 40 (83.3%) |  |
| **COVID-19 characteristics** | | | | |
| Date of first recorded COVID-19 diagnosis (by local peak) |  |  |  | 0.37 |
| February 1, 2020 to September 15, 2020 | 0 (0.0%) | 0 (0.0%) | 0 (0.0%) |  |
| September 16, 2020 to June 20, 2021 | 0 (0.0%) | 0 (0.0%) | 0 (0.0%) |  |
| June 21, 2021 to November 20, 2021 | 86 (10.3%) | 78 (9.9%) | 8 (16.7%) |  |
| November 21, 2021 to April 1, 2022 | 312 (37.3%) | 297 (37.6%) | 15 (31.3%) |  |
| April 2, 2022 to October 15, 2022 | 162 (19.4%) | 150 (19.0%) | 12 (25.0%) |  |
| October 16, 2022 to June 1, 2023 | 101 (12.1%) | 94 (11.9%) | 7 (14.6%) |  |
| June 2, 2023 to November 15, 2023 | 51 (6.1%) | 49 (6.2%) | 2 (4.2%) |  |
| November 16, 2023 to May 1, 2024 | 73 (8.7%) | 72 (9.1%) | 1 (2.1%) |  |
| May 2, 2024 to Dec 15, 2024 | 52 (6.2%) | 49 (6.2%) | 3 (6.3%) |  |
| Status at first recorded COVID-19 diagnostic encounter |  |  |  |  |
| Hospitalized within 30 days | 380 (45.4%) | 355 (45.0%) | 25 (52.1%) | 0.34 |
| Intensive care admission within 30 days | 71 (8.5%) | 62 (7.9%) | 9 (18.8%) | 0.009 |
| Differences across exposure groups compared using Fisher’s exact tests (categorical variables) or Kruskal Wallis tests (continuous variables) Includes SOT recipients who survived at least 90 days following their first recorded COVID-19 laboratory test positive. Exposures: Demographic and clinical variables abstracted from EMR at first COVID-19 diagnostic encounter. Outcome: Long COVID diagnosis determined by presence of U09.9 ICD-10 code. Patients first diagnosed with COVID-19 before October 1, 2021 (the date the code U09.9 was instantiated) and patients with a first recorded COVID-19 laboratory test positive before their most recent transplant were excluded from this analysis. | | | | |
|  |  |  |  |  |
|  |  |  |  |  |

| Table S.1. 2 -year post transplant death/graft failure among solid organ transplant recipients not diagnosed with COVID-19 2020-2024 | | | |
| --- | --- | --- | --- |
|  |  |  |  |
|  | Death/Graft failure- 1 year post transplant | | |
|  | Total N=1,082 | Events n=94 |  |
| **Demographics at first reported COVID-19 episode** | *n (%)* | *n (%)* | p-value |
| Age at transplant (years), median (IQR) | 57 (45-65) | 60 (46-67) | 0.15 |
| Transplanted organ |  |  | 0.012 |
| Kidney | 463 (42.8%) | 35 (37.2%) |  |
| Liver | 436 (40.3%) | 44 (46.8%) |  |
| Heart | 86 (7.9%) | 3 (3.2%) |  |
| Lung | 60 (5.5%) | 11 (11.7%) |  |
| Heart-Lung | 1 (0.1%) | 0 (0.0%) |  |
| Pancreas | 4 (0.4%) | 1 (1.1%) |  |
| Kidney-Pancreas | 32 (3.0%) | 0 (0.0%) |  |
| Other (Includes Islet) | 0 (0.0%) | 0 (0.0%) |  |
| Donor type |  |  | 0.014 |
| Deceased donor (brain death) | 806 (74.5%) | 79 (84.0%) |  |
| Deceased donor (cardiac death) | 117 (10.8%) | 11 (11.7%) |  |
| Living donor | 155 (14.3%) | 4 (4.3%) |  |
| Unknown | 4 (0.4%) | 0 (0.0%) |  |
| Year of most recent transplant |  |  | 0.34 |
| 2020 | 348 (32.2%) | 24 (25.5%) |  |
| 2021 | 194 (17.9%) | 17 (18.1%) |  |
| 2022 | 241 (22.3%) | 27 (28.7%) |  |
| 2023 | 299 (27.6%) | 26 (27.7%) |  |
| Transplant outcome (first recorded event) |  |  | -- |
| Death | 78 (7.2%) | 78 (83.0%) |  |
| Graft failure | 16 (1.5%) | 16 (17.0%) |  |
| Differences across exposure groups compared using Fisher’s exact tests (categorical variables) or Kruskal Wallis tests (continuous variables) Includes patients whose most recent transplant was between January 1, 2020 and December 15, 2023. Exposures: Demographic and clinical variables abstracted from UNOS dataset. Outcome: Composite all-cause mortality or graft failure within 1 year of transplant date. Patients with a transplant date within 1 year (outcome 2) of study end date and those lost to follow-up were excluded.  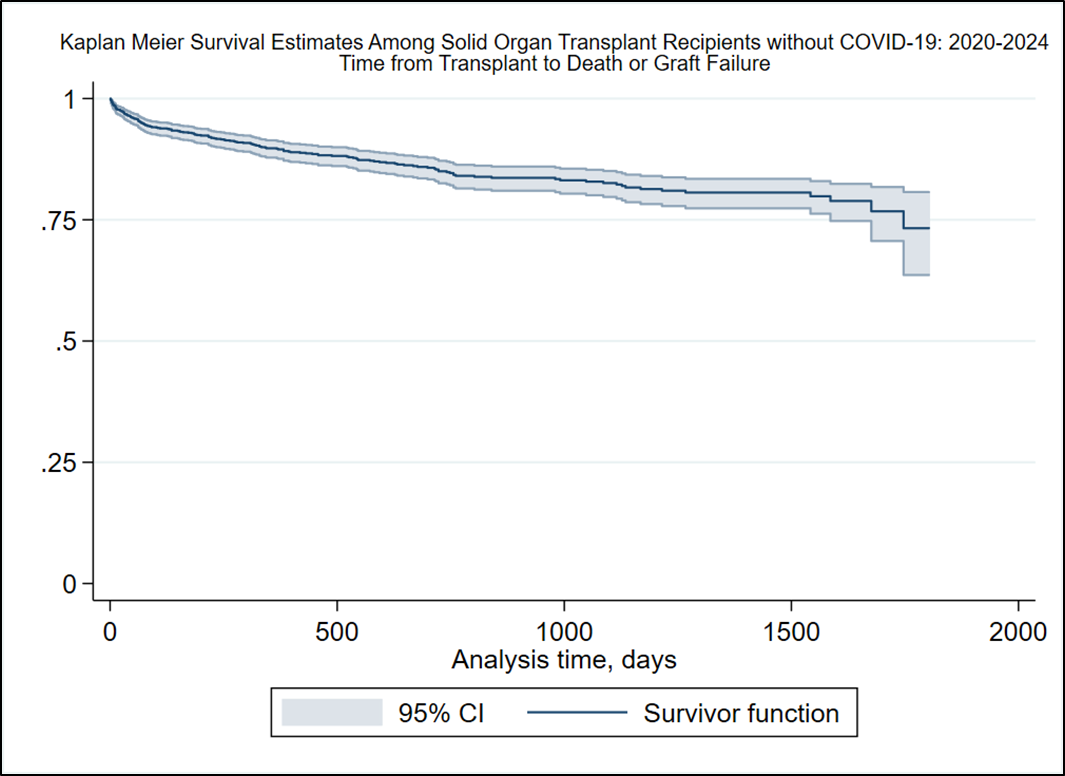 | | | |
|  |  |  |  |
|  |  |  |  |
| \|  \| \| --- \| |  |  |  |

| Table S.3. Composite death/graft failure among solid organ transplant recipients diagnosed with long COVID | | | |
| --- | --- | --- | --- |
|  |  |  |  |
|  | Death/Graft failure- post long COVID diagnosis | | |
|  | Total N=122 | Events n=17 |  |
| **Demographics at first reported COVID-19 episode** | *n (%)* | *n (%)* | p-value |
| Age at encounter (years), median (IQR) | 62 (55-69) | 67 (41-71) | 0.22 |
| Gender |  |  | 0.99 |
| Female | 49 (40.2%) | 7 (41.2%) |  |
| Male | 73 (59.8%) | 10 (58.8%) |  |
| Race/Ethnicity |  |  | 0.92 |
| NON-HISPANIC White | 56 (45.9%) | 9 (52.9%) |  |
| NON-HISPANIC Black | 26 (21.3%) | 4 (23.5%) |  |
| NON-HISPANIC Asian | 4 (3.3%) | 0 (0.0%) |  |
| NON-HISPANIC Hawaiian/Pacific | 0 (0.0%) | 0 (0.0%) |  |
| NON-HISPANIC Native American | 0 (0.0%) | 0 (0.0%) |  |
| NON-HISPANIC Other Race | 0 (0.0%) | 0 (0.0%) |  |
| Hispanic or Latino | 35 (28.7%) | 4 (23.5%) |  |
| Unknown | 1 (0.8%) | 0 (0.0%) |  |
| Area Deprivation Index (state rank) |  |  | 0.22 |
| 1 to 2 (Least deprivation) | 32 (26.2%) | 8 (47.1%) |  |
| 3 to 4 | 30 (24.6%) | 5 (29.4%) |  |
| 5 to 6 | 25 (20.5%) | 3 (17.6%) |  |
| 7 to 8 | 22 (18.0%) | 1 (5.9%) |  |
| 9 to 10 (Most deprivation) | 12 (9.8%) | 0 (0.0%) |  |
| Missing | 1 (0.8%) | 0 (0.0%) |  |
| Financial Class at first COVID-19 diagnosis |  |  | 0.99 |
| Private insurance | 41 (33.6%) | 6 (35.3%) |  |
| Medicare/Medicaid | 78 (63.9%) | 11 (64.7%) |  |
| Self-Pay | 0 (0.0%) | 0 (0.0%) |  |
| Other | 1 (0.8%) | 0 (0.0%) |  |
| Missing | 2 (1.6%) | 0 (0.0%) |  |
| **Clinical history (at first COVID-19 diagnosis)** |  |  |  |
| Body mass index |  |  | 0.83 |
| BMI <=18.5 | 1 (0.8%) | 0 (0.0%) |  |
| BMI 18.5-25 | 17 (13.9%) | 2 (11.8%) |  |
| BMI 25-30 | 27 (22.1%) | 2 (11.8%) |  |
| BMI 30-35 | 11 (9.0%) | 2 (11.8%) |  |
| BMI 35-40 | 4 (3.3%) | 0 (0.0%) |  |
| BMI >40 | 2 (1.6%) | 0 (0.0%) |  |
| Missing | 60 (49.2%) | 11 (64.7%) |  |
| Charlson Comorbidity Index Components |  |  |  |
| Asthma | 24 (19.7%) | 3 (17.6%) | 0.82 |
| COPD | 87 (71.3%) | 14 (82.4%) | 0.28 |
| Tuberculosis | 7 (5.7%) | 0 (0.0%) | 0.27 |
| Myocardial Infarction | 63 (51.6%) | 9 (52.9%) | 0.91 |
| Congestive heart failure | 90 (73.8%) | 13 (76.5%) | 0.79 |
| Peripheral vascular disease | 103 (84.4%) | 16 (94.1%) | 0.23 |
| Cerebrovascular disease | 89 (73.0%) | 13 (76.5%) | 0.72 |
| Diabetes without complications | 100 (82.0%) | 12 (70.6%) | 0.19 |
| Diabetes with complications | 92 (75.4%) | 13 (76.5%) | 0.91 |
| Renal disease | 114 (93.4%) | 17 (100.0%) | 0.24 |
| Peptic ulcer disease | 14 (11.5%) | 3 (17.6%) | 0.39 |
| Mild liver disease | 77 (63.1%) | 12 (70.6%) | 0.49 |
| Moderate to severe liver disease | 23 (18.9%) | 2 (11.8%) | 0.42 |
| Dementia | 4 (3.3%) | 2 (11.8%) | 0.034 |
| Hemiplegia | 12 (9.8%) | 4 (23.5%) | 0.041 |
| Rheumatoid disease | 22 (18.0%) | 4 (23.5%) | 0.53 |
| Cancer | 31 (25.4%) | 4 (23.5%) | 0.85 |
| Metastatic cancer | 15 (12.3%) | 3 (17.6%) | 0.47 |
| HIV/AIDS | 1 (0.8%) | 0 (0.0%) | 0.69 |
| **Transplant characteristics** |  |  |  |
| Transplanted organ |  |  | 0.13 |
| Kidney | 29 (23.8%) | 2 (11.8%) |  |
| Liver | 17 (13.9%) | 2 (11.8%) |  |
| Heart | 24 (19.7%) | 1 (5.9%) |  |
| Lung | 49 (40.2%) | 11 (64.7%) |  |
| Heart-Lung | 2 (1.6%) | 1 (5.9%) |  |
| Pancreas | 0 (0.0%) | 0 (0.0%) |  |
| Kidney-Pancreas | 1 (0.8%) | 0 (0.0%) |  |
| Other (Includes Islet) | 0 (0.0%) | 0 (0.0%) |  |
| Donor type |  |  | 0.22 |
| Deceased donor (brain death) | 102 (83.6%) | 15 (88.2%) |  |
| Deceased donor (cardiac death) | 3 (2.5%) | 0 (0.0%) |  |
| Living donor | 15 (12.3%) | 0 (0.0%) |  |
| Missing | 2 (1.6%) | 2 (11.8%) |  |
| Year of most recent transplant |  |  | 0.67 |
| before 2018 | 51 (41.8%) | 6 (35.3%) |  |
| 2018 | 11 (9.0%) | 1 (5.9%) |  |
| 2019 | 6 (4.9%) | 2 (11.8%) |  |
| 2020 | 15 (12.3%) | 1 (5.9%) |  |
| 2021 | 16 (13.1%) | 4 (23.5%) |  |
| 2022 | 13 (10.7%) | 2 (11.8%) |  |
| 2023 | 8 (6.6%) | 1 (5.9%) |  |
| 2024 | 2 (1.6%) | 0 (0.0%) |  |
| **COVID-19 characteristics** |  |  |  |
| Date of first recorded COVID-19 diagnosis (by local peak) |  |  | 0.73 |
| February 1, 2020 to September 15, 2020 | 10 (8.2%) | 1 (5.9%) |  |
| September 16, 2020 to June 20, 2021 | 38 (31.1%) | 6 (35.3%) |  |
| June 21, 2021 to November 20, 2021 | 17 (13.9%) | 4 (23.5%) |  |
| November 21, 2021 to April 1, 2022 | 20 (16.4%) | 2 (11.8%) |  |
| April 2, 2022 to October 15, 2022 | 18 (14.8%) | 2 (11.8%) |  |
| October 16, 2022 to June 1, 2023 | 11 (9.0%) | 1 (5.9%) |  |
| June 2, 2023 to November 15, 2023 | 2 (1.6%) | 1 (5.9%) |  |
| November 16, 2023 to May 1, 2024 | 2 (1.6%) | 0 (0.0%) |  |
| May 2, 2024 to Dec 15, 2024 | 4 (3.3%) | 0 (0.0%) |  |
| Status at first recorded COVID-19 diagnostic encounter |  |  |  |
| Hospitalized within 30 days | 55 (45.1%) | 6 (35.3%) | 0.38 |
| Intensive care admission within 30 days | 17 (13.9%) | 1 (5.9%) | 0.3 |
| Time from long COVID to event: days, median (IQR) |  | 291 (89-345) | -- |
| Transplant outcome (first recorded event) |  |  | -- |
| Death | 15 (12.3%) | 15 (88.2%) |  |
| Graft failure | 2 (1.6%) | 2 (11.8%) |  |
| Differences across exposure groups compared using Fisher’s exact tests (categorical variables) or Kruskal Wallis tests (continuous variables) Includes patients who were diagnosed with long COVID while under observation following a solid organ transplant. Exposures: Demographic and clinical variables abstracted from EHR. Outcome: Composite all-cause mortality or graft failure anytime following transplant from UNOS dataset. Patients no longer being followed at time of long COVID diagnosis were excluded. | | | |
